# Scale‐Dependent Effects of Landscape Heterogeneity on Butterfly Functional and Taxonomic Diversity in Andean Urban Parks

**DOI:** 10.1002/ece3.72341

**Published:** 2025-10-20

**Authors:** Nathali Coral‐Acosta, John Harold Castaño, Darly Tatiana Rodríguez Jiménez, J. Nicolás Urbina‐Cardona

**Affiliations:** ^1^ Departamento de Ecología y Territorio, Facultad de Estudios Ambientales y Rurales Pontificia Universidad Javeriana Bogotá Colombia; ^2^ Grupo de investigación en Biología de la Conservación y Biotecnología Corporación Universitaria de Santa Rosa de Cabal – UNISARC Risaralda Colombia; ^3^ Facultad de Ingeniería y Ciencias Básicas Universidad Central Bogotá Colombia

**Keywords:** functional traits, habitat parameters, landscape metrics, scale of effect, species richness, urban ecology

## Abstract

Urbanization poses a significant threat to biodiversity, reducing native species diversity in cities. Urban green spaces, like parks, become essential refuges for species adapting to altered environments, influenced by anthropogenic infrastructure that shapes species' functional responses. This study evaluates the associations between local vegetation structure, landscape heterogeneity, and butterfly taxonomic and functional diversity in 15 urban parks within the Andean city of Bogotá, Colombia. We measured 15 local‐scale variables and 8 landscape heterogeneity variables at each spatial scale, examining their associations within areas ranging from 250 to 1000 m around the parks. Tree height was positively associated with functional dispersion at the local scale. At smaller landscape scales (250–500 m), species richness was positively associated with the total area of neighborhood (0.1–1.5 ha) and pocket (< 0.1 ha) parks. At larger scales (750–1000 m), functional divergence was correlated with the number of trees, while functional richness and originality were linked to proximity to water bodies. Additionally, fourth‐corner and RLQ analyses revealed a scale‐dependent pattern: At the local scale, tree height and diameter at breast height (DBH) were associated with wing span. At intermediate scales (250–750 m), wing span was also related to the distance from bodies of water, while at 1000 m, the total area of neighborhood parks emerged as an additional factor influencing wing span. Urban management should prioritize a great diversity of tall native trees to support diverse butterfly assemblages at local scales. Enhancing connectivity between pocket and neighborhood parks, integrating water bodies, and increasing the number of native trees within a 750 m radius can significantly boost both taxonomic and functional diversity in Bogotá.

## Graphical abstract

Local vegetation structure and landscape composition significantly affect butterfly diversity, underscoring the role of habitat quality in shaping butterfly assemblages

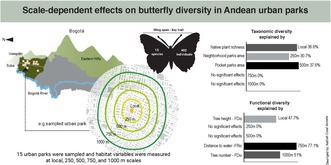

## RESUMEN

La urbanización constituye una amenaza significativa para la biodiversidad al reducir la diversidad de especies nativas en las ciudades. Los espacios verdes urbanos, particularmente los parques, emergen como refugios esenciales para especies adaptadas a entornos alterados, influenciados por la infraestructura antrópica que modula las respuestas funcionales de las especies. Este estudio evalúa las asociaciones entre la estructura de la vegetación local, la heterogeneidad del paisaje y la diversidad taxonómica y funcional de mariposas en 15 parques urbanos de Bogotá, en los Andes de Colombia. Se midieron 15 variables a escala local y 8 variables de heterogeneidad del paisaje, examinando asociaciones en áreas de 250 a 1000 metros alrededor de los parques estudiados. Los resultados revelan patrones dependientes de la escala espacial. A nivel local, la altura de los árboles se asoció positivamente con la dispersión funcional. En escalas pequeñas (250–500 m), la riqueza de especies se relacionó positivamente con el área de parques vecinales (0,1–1,5 ha) y de bolsillo (< 0,1 ha). A escalas mayores (750–1000 m), la divergencia funcional se correlacionó con el número de árboles, mientras que la riqueza y originalidad funcional se vincularon con la proximidad a cuerpos de agua. Los análisis de cuarta esquina y RLQ revelaron patrones específicos dependientes de la escala: a escala local, la altura de árboles y el diámetro a la altura del pecho (DAP) se asociaron con la envergadura alar. En escalas intermedias (250‐750 m), la envergadura alar se relacionó también con la distancia a cuerpos de agua, mientras que a 1000 m, el área de parques vecinales emergió como factor adicional. La gestión urbana debe priorizar una alta diversidad de árboles nativos de gran altura para apoyar ensamblajes diversos de mariposas a escala local. Se recomienda mejorar la conectividad entre parques de bolsillo y vecinales, integrar cuerpos de agua y aumentar el número de árboles nativos dentro de un radio de 750 m. Estas medidas pueden incrementar significativamente la diversidad taxonómica y funcional de mariposas en Bogotá.

## 1 Introduction

Urbanization, along with agricultural intensification, is a major driver of biodiversity loss, particularly in cities surrounded by agricultural areas that further degrade natural habitats (Grimm et al. 2008). The urban matrix, dominated by artificial surfaces like roads and housing, significantly reduces native biodiversity (Aronson et al. 2017). Urban green spaces such as parks and gardens represent crucial refuges for native species within cities despite being isolated patches in the urban landscape, often dominated by exotic and invasive plant species (McKinney 2008). Poor connectivity between these areas necessitates improving the urban matrix quality (Lovell and Johnston 2009). The degradation of these green spaces leads to homogenization of insect assemblages and reduced species abundance (McIntyre 2000), underscoring their importance as the remaining habitats provide resources and shelter for surviving native species (Aronson et al. 2017). However, how species respond to these anthropogenic ecosystems depends on the morphological and physiological traits of each species, to take advantage of habitat resources for their survival (Han et al. 2021). Functional traits are morphological, physiological, phenological, or behavioral characteristics measurable at the individual level that influence a species' fitness and mediate its response to environmental filters, thereby shaping community structure and ecosystem functioning (Díaz et al. 2013; Mouillot et al. 2013).

Urban animal diversity depends on the size, quantity, and quality of green areas, where quality refers to a higher proportion of native species relative to exotic or invasive ones (Lepczyk et al. 2017). Habitat quality is also influenced by the presence of water bodies, which act as landscape‐structuring elements that benefit butterflies both directly and indirectly. Water bodies provide essential resources for thermoregulation and reproduction while indirectly maintaining humid microhabitats that support nectar‐producing plants (Brown and Freitas 2002; Gandhi and Kumar 2015). Additionally, watering practices among green areas (i.e., gardens) and both natural and artificial water sources available in cities enable the development of flowering plants around them, increasing the availability of nectar resources for pollinators such as butterflies (Lizée et al. 2011; Biella et al. 2022). This hydroecological connectivity is particularly important in urban environments where fragmentation limits access to natural water resources. More specifically, the abundance and richness of pollinating insects depend on the proximity and size of domestic gardens and wooded parks that provide floral resources (Matteson and Langellotto 2010; Levé et al. 2019). While generalist species may thrive in urban environments, forest specialists—particularly those with limited dispersal capacity—struggle to establish viable populations due to the scarcity and fragmentation of suitable habitats (Kowarik 2011; Koh and Sodhi 2004). Understanding these dynamics is essential for designing effective conservation plans that support diverse ecological strategies (Gaston 2010). In particular, promoting connectivity between habitat patches may mitigate the isolation of sedentary species, such as some specialist butterflies, by facilitating dispersal and gene flow (Pla‐Narbona et al. 2022). This underscores the importance of considering the spatial configuration and ecological quality of green areas when developing local‐scale urban conservation strategies (Grimm et al. 2008).

One approach to estimate potential relationships between species diversity and the surrounding landscape heterogeneity is to measure land cover type area and other metrics, such as distance to bodies of water, at specific distances from the species diversity sampling sites (also referred to as the focal patch). Then, identify the buffer distances at which statistical models most supported explain these relationships, showing a biologically meaningful scale on species diversity (*scale of effect*; Jackson and Fahrig 2012). Understanding the scale of effect for a biological variable such as species richness or species abundance is crucial for accurately assessing ecological dynamics and informing effective conservation strategies. Identifying the appropriate spatial scale allows researchers to determine how landscape features are associated with biological patterns and processes, thereby enabling more precise predictions and targeted management actions to preserve biodiversity.

However, the scale of effect for butterflies remains understudied, and the limited existing research shows varying results. Flick et al. (2012) found that in an agricultural landscape, the scale of effect on butterfly richness and abundance was most significant within 250 m of the sampling transect. Other studies suggest that a 320‐m radius scale is crucial for interpatch movements of common macromoth species in agricultural landscapes (Merckx et al. 2009, 2019). In contrast, additional research indicates that the scale of effect related to landscape structure varies across the density and richness of plant functional traits associated with pollination and seed dispersal syndromes, with most variables showing relationships at spatial scales greater than 750 m (Martello et al. 2023). Therefore, when analyzing landscape effects, it is essential to assess each taxonomic group at its biologically relevant spatial scale, as different groups respond differently to landscape heterogeneity (Jackson and Fahrig 2012; Moraga et al. 2019).

Studies of urban biodiversity have focused on describing the taxonomic diversity of assemblages using the metrics of richness, dominance, and evenness, assuming that all species are ecologically equivalent and where their abundances determine their relative importance in the ecosystems (McKinney 2008; Mouillot et al. 2013). Consequently, it is crucial to consider the species' identity within the assemblage by analyzing functional diversity. This facet of diversity assesses the range, distribution, and value of functional traits directly influencing organism performance (Díaz et al. 2013; Mouillot et al. 2013). Such analysis of functional diversity in urban green areas allows evaluating not only the taxonomic diversity of assemblages but also understanding the functional responses of species to changes in landscape heterogeneity surrounding their habitat in an urban context (Moraga et al. 2019). Therefore, analyzing functional diversity becomes critical for understanding community assembly processes and ecosystems functioning in urban landscapes, providing insights that taxonomic diversity alone cannot describe.

In this study, butterflies were used as a model due to the stability and clarity of their taxonomic classification (Bonebrake et al. 2010), the large amount of information on their response to environmental disturbances (Koh and Sodhi 2004; Clark et al. 2007), their ability to inhabit urban environments, and their role in ecosystem service provision (Ramírez‐Restrepo and Macgregorfors 2017). Moreover, butterflies respond to the composition and configuration of the landscape at different spatial scales (Flick et al. 2012; Bergman et al. 2018). Consequently, butterflies are an excellent model group to study the patterns and ecological processes in urban parks (or green urban areas) and their responses to the environmental filters imposed by landscape heterogeneity (Lizee et al. 2016). Specialist species, like forest butterflies, struggle to survive in urban areas due to the lack of suitable and continuous habitats, making them good indicators for high‐value habitats (Kowarik 2011; Gaston 2010). Additionally, being charismatic organisms, they can be used for educational purposes that allow people to participate in management actions of urban green areas and to increase biophilic values (Ramírez‐Restrepo and Macgregorfors 2017).

The city of Bogotá, with its complex Andean topography, altitudinal range, and heterogeneous matrix of natural and anthropogenic green spaces, offers a particularly relevant setting for evaluating scale‐dependent ecological responses in tropical urban environments. The main objective of this research was to evaluate the associations between local vegetation structure and the surrounding landscape heterogeneity with the taxonomic and functional diversity of butterflies in urban parks of Bogotá. This study aimed to identify the spatial scales at which these factors show the strongest associations and to inform habitat management strategies for butterflies in urban environments. We hypothesize that local vegetation structure variables and landscape heterogeneity metrics will be significantly associated with variations in the taxonomic and functional diversity of butterflies in urban parks, consistent with previous studies highlighting the influence of habitat quality and landscape context on urban biodiversity (Lepczyk et al. 2017; Moraga et al. 2019). We expect that the strength and direction of these effects will vary depending on the spatial scale considered (e.g., local scale within parks vs. landscape scale around parks), in line with the scale‐dependent ecological responses documented in butterflies (Flick et al. 2012; Bergman et al. 2018). Specifically, we anticipate that taxonomic and functional richness will be higher in parks with greater native plant richness, taller trees, and larger park areas at local scales, reflecting findings on habitat complexity and resource availability enhancing butterfly diversity (Lepczyk et al. 2017; Moraga et al. 2019). Conversely, we expect that a higher proportion of built‐up areas around parks will lead to lower species richness and a less diverse butterfly assemblage at the 250‐m scale, consistent with urban homogenization and biotic filtering effects (McKinney 2008; Kowarik 2011). We predict that the extent of built‐up areas and the distance from water bodies will be negatively associated with functional diversity, particularly at the 750‐m scale (scale of effect), reflecting scale‐dependent impacts of landscape heterogeneity on butterfly functional traits (Flick et al. 2012; Bergman et al. 2018). However, at the 1000‐m scale, we anticipate that these effects will be insignificant due to the limited dispersal capacity of specialist and sedentary butterflies within the assemblage (Pla‐Narbona et al. 2022).

## 2 Materials and Methods

### 2.1 Study Area

**FIGURE 1:**
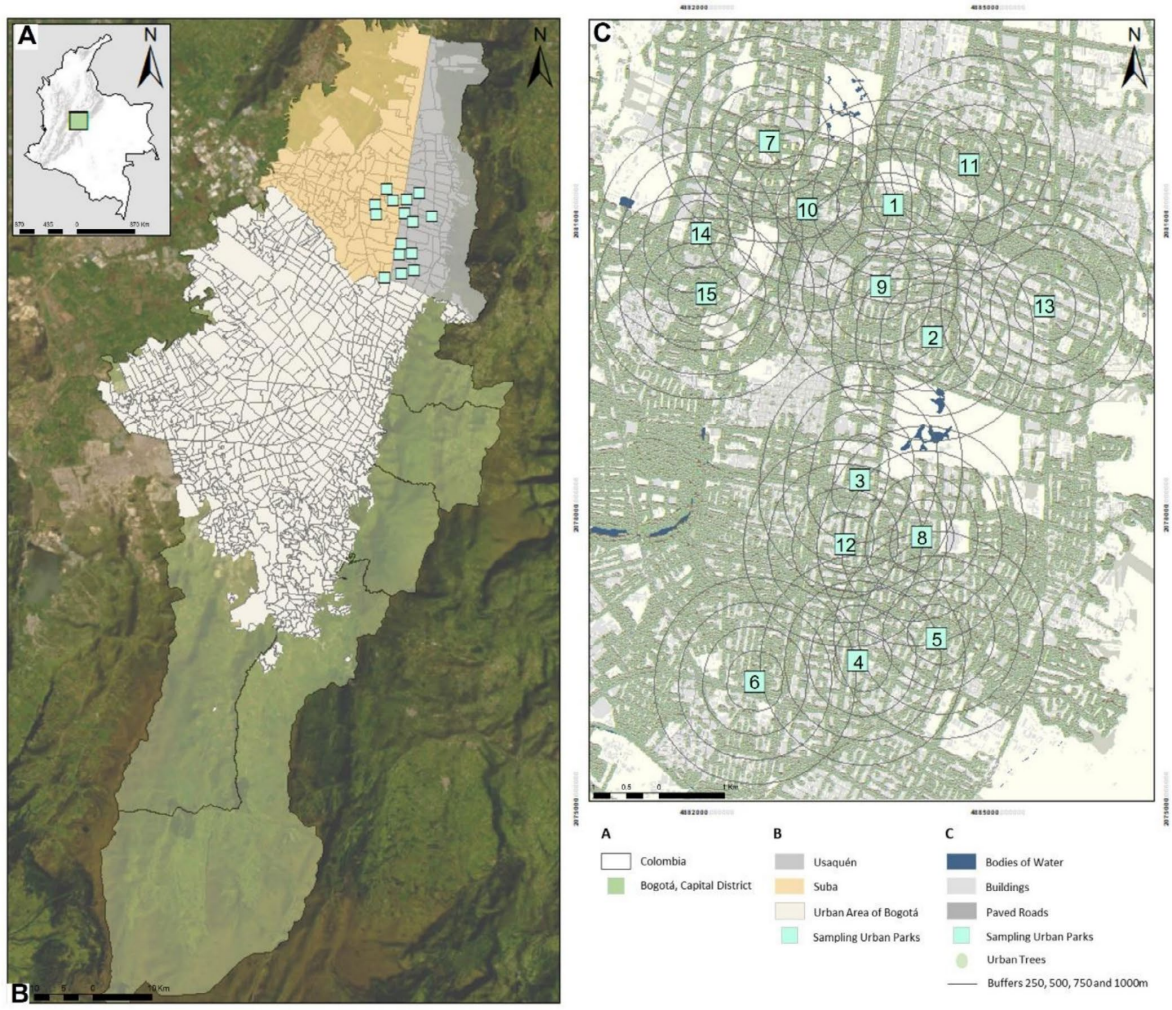
(A) Location of the Capital District, Bogotá in Colombia. (B) Localities of Usaquén and Suba to the north of the city are indicated in gray and yellow tones, respectively. (C) Spatial distribution of the 15 urban parks sampled between the localities of Usaquén and Suba in the city of Bogotá.

This study was carried out in the city of Bogotá, Colombia, located at 2650 m a.s.l. in the Eastern Cordillera of the Andes. The city has approximately 7.5 million inhabitants and is the largest city in the country with an area of 163,635.07 ha, of which 23.2% of the space corresponds to the urban area; the other percentage corresponds to rural areas with a high dominance (50.8%) of páramo ecosystems, 11.8% of pastures for livestock, and 4.5% of Andean cloud forests (Secretaría Distrital de Ambiente (SDA) and Conservación Internacional (CI) 2010). It is delimited to the east by the Eastern Hills (or Cerros Orientales), characterized by a mosaic of native Andean Forest vegetation mixed with forest plantations of exotic species (*Eucaliptus* spp., *Pinus* spp.), pastures for livestock, and mining quarries for construction materials; and to the west by the Bogotá River (Secretaría Distrital de Planeación (SDP) 2020). The Ecological Main Structure (EMS) is a network of spaces and corridors that support biodiversity and ecological processes while accommodating human activities (SDP 2020). As a planning tool, it aimed to provide environmental services for sustainable development and includes both natural and seminatural ecosystems. In Bogotá, the EMS connects the city with periurban and rural areas through wetlands, protected areas, and various parks and greenways that enhance ecological connectivity (Andrade et al. 2013). This system helps maintain biodiversity and ecological integrity, supporting social and economic development in urban and rural areas (SDA and CI 2010). Of the 21 localities in which the city of Bogotá is divided, Suba (4.7208°N, 74.0748°W) and Usaquén (4.7033°N, 74.0329°W) (Figure 1), located to the north, have the best environmental quality due to the greater number of urban trees and the high number of urban parks (SDP 2020). Most of these parks correspond to “neighborhood parks” that are characterized as green areas for passive recreation whose purpose is the social integration of local people (SDP 2020). The Secretaría Distrital de Planeación (SDP 2020) classifies urban parks into: (1) pocket parks: < 0.1 ha, intended for passive recreation and contribute to environmental control and urban tree planting; (2) neighborhood parks: most abundant in the city, present between 0.1 ha and approximately 1.5 ha, intended for community entertainment and gatherings; (3) zonal parks: they present between 1.5 and 10 ha, intended for active recreation with specialized elements for sports; (4) metropolitan parks: more than 10 ha, intended for the generation of landscape values and both active and passive recreation activities. These last two types of parks are part of the main ecological structure of the city (SDP 2020).

Bogotá is also characterized by having only 4.9% of public green areas within the urban zone, making it one of the cities with the most limited per capita access to public green spaces globally (Vidal et al. 2020). However, its suburban and periurban areas support high biodiversity, including some of the highest bird species richness reported among major cities worldwide. This biodiversity is supported by a mosaic of natural land cover types (e.g., wetlands and Andean forests) and anthropogenic green spaces that provide habitat for native fauna such as butterflies (Caicedo‐Hernández et al. 2017; Durán‐Prieto and Molina‐Fonseca 2020).

### 2.2 Study Design

In this study, we selected 15 neighborhood parks, located in Suba and Usaquén localities in northern Bogotá (Figure 1; Table S1). The parks sampled are characterized by having an average canopy height of 9.84 m, 47.14% tree cover, and up to 14 species of native plants. However, these parks are not considered part of the main ecological structure of the city (SDP 2020). To ensure the spatial independence of the butterfly samples, the selected parks were separated from each other by at least 700 m (mean = 3700 m; min = 700; max = 11030 m) following Freitas et al. (2021). In the parks, tree and shrub pruning is carried out by the local government, and leaf litter is collected in six of the parks. Based on studies by Matteson and Langellotto (2010) and Levé et al. (2019), we defined five spatial scales of influence in the parks for this study: local (focal patch inside each park) and radii of 250, 500, 750, and 1000 m around each focal patch (Figure S1). *Local variables* were considered metrics that were measured within each park (Matteson and Langellotto 2010), and *landscape variables* were metrics recorded around each focal patch and within each of the areas of influence (Jackson and Fahrig 2012). For the design of the study, we locally categorized the parks based on 15 variables explained in section “Habitat variables at local and landscape scales” (Table S2).

### 2.3 Butterfly Sampling

To sample the 15 parks, three field trips were conducted in September 2021, November 2021, and January 2022. Butterfly collection in each park was performed using the direct search method with an entomological net, systematically covering the entire park without repeating routes. Additionally, in each park, two van Sommeren Rydon traps were set approximately 100 m apart and 1.5 m above ground, baited with decomposing shrimp, human feces, and fermented bananas (Freitas et al. 2021). All nonbutterfly insects caught in the traps were released in situ. During each field trip, the traps were active for 8 h, and sampling was conducted by two people between 07:00 and 15:00, totaling 1440 sampling hours. The captured specimens were sacrificed and stored in Milano paper envelopes for later identification and analysis at the Functional Ecology Laboratory of the Pontificia Universidad Javeriana. Once prepared and identified, the specimens were deposited in the Entomology Collection of the Pontificia Universidad Javeriana (MPUJ‐ENT). Taxonomic keys, illustrated guides, and checklists from DeVries (1987) and Garwood et al. (2021) were used for butterfly species identification.

### 2.4 Measurement of Functional Traits

Each specimen collected was photographed dorsally and ventrally (Figure S2). Based on the standardized measurement protocol for insect functional traits (Moretti et al. 2017), 13 functional traits were selected (See Appendix S1) and were grouped into two categories: (1) body morphology traits, since they are related to the strategy of obtaining resources and allow understanding the responses of species to changes in the environmental conditions of their habitat (Moretti et al. 2017); and (2) color of the individuals, since biotic and abiotic interactions have an effect on pigmentation and influence the *fitness* of organisms (Moretti et al. 2017). Functional traits were measured in 268 individuals across the 15 sampled species, adhering to the sampling permit requirement of collecting no more than five individuals per species per park per field trip. See Appendix S1 for a full description of the trait measurement methods.

### 2.5 Habitat Variables at Local and Landscape Scales

At the local scale, 11 variables that characterize the structure of the vegetation were measured in the total area of each park: (1) the number of trees, determined by making direct counts of all the individuals in each park; (2) the average height of the trees; (3) the average height of the stem, measured with the “Forest Clinometer‐2019” version 1.5 for SO‐Android (iTech Desenvolvimentos 2020); (4) the average diameter at breast height—DBP; (5) the average crown diameter, measured directly from each individual using a decameter; (6) the average number of flowers per tree, obtained by direct counts on each individual; (7) the area of planted gardens within parks; (8) the average number of flowers present in the gardens, directly measured for each garden; (9) the richness of plant species; (10) the richness of native species; and (11) the richness of nonnative species directly counted through taxonomic identification of all the individuals present in each park. Additionally, four variables were obtained from the official page of the government of the city of Bogotá (https://datosabiertos.bogota.gov.co/) and analyzed using the “Clipping Masks” method in ArcGIS 10.7.1 software. We calculated the following variables for each sampled urban park: (12) total park area, (13) area of tree cover within the park, (14) paved coverage area in the surrounding area (e.g., adjacent roads, parking lots, and other constructions), and (15) area of soft cover (e.g., grass). At the landscape scales (areas of influence of 250, 500, 750, and 1000 m around each focal patch), eight variables were obtained from the official page of the government of the city of Bogotá (https://datosabiertos.bogota.gov.co/) and were measured using the “Clipping Masks” method in ArcGIS 10.7.1 software: (1) number of trees, (2) total area of neighborhood parks, (3) total area of pocket parks, (4) total area of zonal parks, (5) total area of metropolitan parks, (6) total built‐up area, (7) total area of paved roads, and (8) average distance to bodies of water was calculated. These spatial scales (250, 500, 750, 1000 m) were selected based on butterfly dispersal abilities and the typical range of species movements within urban landscapes (Gutiérrez et al. 1999; Wood and Pullin 2002).

## 3 Analysis of Data

### 3.1 Taxonomic Diversity of Butterfly Assemblages in Urban Parks

For each park, we estimated the taxonomic alpha diversity index expressed in terms of effective numbers of species (Jost 2006) and the representativeness of the samplings. The exponent *q* indicates the number of species that are registered in the sample considering their relative abundances (Chao et al. 2016). Therefore, the diversity of order *q* = 0 is independent of the abundances of the species, describing the species richness (Chao et al. 2016). Additionally, the abundance of butterfly species (previously transformed to square root) from each park was utilized to create a Bray–Curtis similarity matrix to describe the assemblage structure. These analyses were performed using the online version of iNEXT developed by Chao et al. (2016). We focus primarily on species richness and assemblage structure as key metrics for assessing both alpha and beta diversity, which provide insights into the competitive dynamics within urban butterfly assemblages.

Finally, we used the 9999 resampling of species richness with the Bootstrap estimator and their 95% confidence intervals to evaluate the completeness of the species inventory for each park, determining if the sampling was sufficient. The Bootstrap resampling technique generates multiple datasets from partial data to estimate total species richness and calculates confidence intervals to assess how close the observed species numbers are to the true total (Chao et al. 2016).

### 3.2 Functional Diversity of Butterfly Assemblages in Urban Parks

A Spearman correlation analysis was performed to identify traits that presented collinearity (values above 0.70) to discard them for subsequent analyses (Figure S3). This analysis was performed using the pairs.panels function from the R package Psych version 2.5.6 using R version 4.3.1 (R Core Team 2020; Revelle 2025). Of the 13 functional traits measured, the wingspan and thorax width were not collinear with any other trait; therefore, functional diversity indices were calculated from these two (Figure S3).

We calculated four functional diversity indices using the alpha.fd.multidim function in the mFD package (Magneville et al. 2021): (1) functional richness (Fric), which represents the amount of functional space occupied by the species in an assemblage; (2) functional dispersion (FDis), which is the average distance that the individuals of the species have towards the centroid of all the species present in an assemblage; (3) functional divergence (FDiv), which indicates the distribution of abundances within a functional space occupied by the species; and (4) functional originality (FOri), which identifies the isolation of species in the functional space of a community (Laliberté and Legendre 2010; Mouillot et al. 2013). To assess functional diversity, we focused on these four indices as they collectively capture different aspects of species' ecological roles and responses to environmental gradients (Magneville et al. 2021).

### 3.3 Relationship Between Taxonomic and Functional Diversity of Butterfly Assemblages and Environmental Variables

We conducted a Spearman correlation analysis of local vegetation structure and landscape heterogeneity metrics for each of the four areas of influence to identify collinear variables (Tables S2–S6). Variables with values exceeding 0.7 were excluded from subsequent analyses. In this context, the models explaining changes in response variables (diversity indices) were constructed using up to four predictor variables at each spatial scale. To identify the variables associated with variation in the taxonomic and functional facets of butterfly diversity, distance‐based linear models (DistLM; Legendre and Anderson 1999) were assessed at each spatial scale, ranging from the local scale to the landscape scales of influence (250, 500, 750, and 1000 m around the focal patches), following the approach of Desaegher et al. (2022).

In terms of taxonomic diversity, the response variables included assemblage structure (Bray–Curtis similarity) and alpha diversity (Euclidean distance matrices for species richness of order *q* = 0). For functional diversity, the response variables consisted of Euclidean distance matrices for each of the four previously described indices.

For each spatial scale, we generated an Euclidean distance matrix from a scale‐specific set of previously standardized predictor variables. Using the BEST subroutine in DISTLM, we explored all possible combinations of noncollinear predictor variables and computed the Akaike Information Criterion corrected for small sample sizes (AICc) for each candidate model (Legendre and Anderson 1999; Johnson and Omland 2004). Model selection was based on the AICc, which estimates relative prediction error by balancing model complexity and fit, penalizing overly complex models (Sutherland et al. 2023). Lower AICc values indicate models with less expected information loss compared to competing models but do not necessarily reflect the best absolute fit (Aho et al. 2014; Tredennick et al. 2021). Once the most supported model was chosen, its significance was evaluated using a Pseudo‐*F* test and its associated *p*‐perm value. For each significant predictor variable in the most supported model, we reported the sum of squares, Pseudo‐*F* test, *p*‐perm value, and the proportion of variance explained. These analyses were conducted using the PRIMER v7 package and the PERMANOVA add‐on v1.0.4 (Anderson et al. 2008; Clarke and Gorley 2015).

Furthermore, to visualize the ordering patterns in multivariate response variables such as assemblage structure (using the Bray–Curtis similarity index), we conducted a distance‐based redundancy analysis (dbRDA) using the Legendre and Anderson (1999) subroutine. Specifically, we visualized vectors associated with predictor variables that exhibited a Pearson correlation value > 0.5 with the first two axes of the dbRDA. These analyses were conducted using the PRIMER v7 package and the PERMANOVA add‐on v1.0.4 (Anderson et al. 2008; Clarke and Gorley 2015).

Finally, to assess the relationship between traits and environmental variables, a fourth‐corner analysis was performed, followed by the RLQ method using the “ade4” package in R (Bougeard and Dray 2018; Dray and Dufour 2007; Dray et al. 2014). The RLQ analysis integrates three datasets: environmental variables (*R*), species abundance (*L*), and species traits (*Q*). This method identifies linear combinations of environmental variables and species traits that maximize their covariance, weighted by species abundance. RLQ provides a multivariate approach to assess trait–environment relationships across multiple traits and environmental gradients simultaneously (Dray and Legendre 2008).

Fourth‐corner analysis complements RLQ by testing the significance of associations between individual traits and environmental variables using permutation procedures. As recommended by Dray et al. (2014), we applied a two‐step testing procedure to control for Type I error rates. Specifically, Model 4 tests for associations between traits and environmental variables, considering species abundance as a weighting factor. Model 5 assesses overall significance by combining results from multiple trait–environment associations, providing a global test of the hypothesis that species traits are not randomly associated with environmental gradients. By combining RLQ and Fourth‐Corner methods, we identified both global patterns (via RLQ) and specific trait–environment associations (via Fourth‐Corner). This integrative approach provides complementary insights, ensuring robust inference on the ecological processes underlying butterfly trait responses to urbanization.

## 4 Results

### 4.1 Taxonomic Diversity of Butterfly Assemblages in Urban Parks

According to the bootstrap estimator, the completeness of the samplings in all the parks fluctuated between 85% and 100%, indicating that the sampling is an adequate representation of the butterflies present in the studied urban parks (Table S7). We collected a total of 402 individuals of 15 species of butterflies belonging to three families (Table S8). Species richness fluctuated between 5 and 7 species per park (Table S9). Pieridae was the family with the highest number of species (47%), followed by Nymphalidae (33%) and Hesperiidae (20%). The species with the highest number of individuals were *Leptophobia aripa aripa*, followed by *Colias dimera* and *Eantis pallida*, which were registered in most of the sampled urban parks. In contrast, 7 of the 15 species ( *Hylephila phyleus phyleus* , *Telegonus alector alector*, *Aeria eurymedia*, *Heliconius erato hydara*, *Glutophrissa drusilla drusilla*, *Leptophobia eleone eleone*, and *Tatochila xanthodice*) had only one individual.

The largest park (P7), located to the northwest in Sector 1 of the sampling area (Table S1), had the highest number of rare species (*T. alector alector*, *A. eurimedia*, and *T. xanthodice*) (Table S8). It should be emphasized that the species *A. eurimedia*, *G. drusilla drusilla*, and *H. erato hydara* corresponded to new geographical distribution records for the urban area of Bogotá; as these species inhabit lower elevations (Andrade‐C et al. 2007; DeVries 1987; Le Crom et al. 2004).

### 4.2 Relationship Between Taxonomic and Functional Indices of Butterfly Diversity and Local and Landscape Variables

At the local scale, tree height explained 25.1% of the variation in butterfly assemblage structure, while native plant richness accounted for 36.58% of the changes in species richness (Table 1; Appendix S2; Table S10). dbRDA identified three additional significant predictors: Average DBP (m), average number of flowers per tree, and soft cover area (%). At the 250 m scale, distance to bodies of water explained 18% of the variation in assemblage structure, while neighborhood parks area explained 30.7% of richness (Table 1; Appendix S2; Table S10). dbRDA revealed three important predictors: Pocket parks area, built‐up area, and paved roads area. At the 500 m scale, neighborhood park area explained 23.6% of variation in assemblage structure, while pocket parks area accounted for 37.6% of richness (Table 1; Appendix S2; Table S10). dbRDA identified two key predictors: Number of trees and pocket parks area. At the 750 m scale, no landscape variables significantly explained assemblage structure or alpha diversity indices, but dbRDA identified three additional predictors: Pocket parks area, zonal parks area, and metropolitan parks area. At the 1000 m scale, dbRDA identified three important predictors: Number of trees, neighborhood parks area, and zonal parks area.

**TABLE 1:** Significant predictor variables and their explained variation (%) in taxonomic and functional diversity indices across five spatial scales of influence (ranging from local up to 1000 m around the focal patches) using the most supported models adjusted through the DistLM routine.

Facet of diversity	Diversity indices	Local scale (*R* ^2^)	250 m scale (*R* ^2^)	500 m scale (*R* ^2^)	750 m scale (*R* ^2^)	1000 m scale (*R* ^2^)
Taxonomic diversity	Richness (*q* = 0)	Richness of native plant species (36.58%)	Total area of neighborhood Parks (30.7%)	Total area of pocket Parks (37.6%)		
Functional diversity	Fdis	Average height of Trees (47.7%)				
	Fric				Average distance to bodies of Water (77.1%)	
	Fdiv				Number of trees (51.7%)	Number of trees (51%)
	Fori				Average distance to bodies of Water (46.2%)	

For functional diversity, tree height most supported variation in FDis at the local scale (*R* ^2^ = 47.7%) (Table 1; Appendix S2; Table S10). dbRDA identified five key predictors for functional diversity: Average tree height, paved coverage area, average DBP, richness of nonnative species, and richness of native species. No variables significantly explained functional diversity indices at the 250 m or 500 m scales, but dbRDA identified important predictors for functional diversity at the 250 m scale (number of trees, average distance to bodies of water) and at the 500 m scale (total area of pocket parks). At the 750 m scale, variations in functional diversity indices, including FRic and FOri, were explained by the average distance to bodies of water (*R* ^2^ = 26.5%, 77.1%, and 46.2%, respectively) (Table 1; Appendix S2; Table S10), while FDiv was associated with the number of trees (*R* ^2^ = 51.7%) (Table 1; Appendix S2; Table S10). dbRDA identified four important predictors for functional diversity at the 750 m scale (Appendix S3): Neighborhood parks area, pocket parks Area, zonal parks area, and metropolitan parks area. Finally, at the 1000 m scale, FDiv variation was explained by the number of trees (*R* ^2^ = 51%) (Table 1; Appendix S2; Table S10). dbRDA identified three key predictors for functional diversity at this scale: Number of trees, built‐up area, and paved roads area.

The fourth‐corner analysis enabled the identification of environmental variables showing significant correlations with butterfly functional traits at each scale. At the local scale, Model 5 revealed significant positive correlations between the average tree height and the average DBH with butterfly wing span (Table 2; Table S11). At the 250 m scale surrounding the sampled parks, no model showed statistically significant *p* values between environmental variables and butterfly functional traits. However, in Model 4, the relationship between average distance to water bodies and wing span was identified as positive and marginally nonsignificant (Table 2; Table S11). At the 500 m scale, Model 5 identified the most significant correlations between the environmental variables of tree number (negative) and average distance to water bodies (positive) with wing span (Table 2; Table S11). At the 750 m scale, Model 5 identified significant positive correlations between distance to water bodies and wing span (Table 2; Table S11), and at the 1000 m scale, Model 5 also identified significant correlations (negative) between the total area of neighborhood parks and wing span (Table 2; Table S11). Notably, Model 5 (which integrates environmental variables with species' traits) consistently provided the most significant correlations across most scales (except 250 m). Furthermore, butterfly wing span was the most correlated trait with environmental variables across landscape scales considered in this study. RLQ analysis revealed that the first axis exhibited a projected inertia ranging from 65.46% at the local scale to 91.76% at the 500 m scale, capturing the majority of the relationships between environmental variables and species traits. Detailed results are provided in Table S12 and Appendix S4.

**TABLE 2:** Summary of fourth‐corner analysis results for five spatial scales (local, 250, 500, 750, and 1000 m around the focal sampling patch), with Model 5 (restricted by an environmental gradient) as the most significant for four scales and Model 4 (permuted within site groups) for the 250 m scale.

Scale	Best model	Fourth‐corner analysis			
		Variables	Obs	Std. Obs	*p*
Local	5th	Average Height of Trees/WingSpanmm	*r* = 0.2674	2.9069	0.004
		Average DBP/WingSpanmm	*r* = 0.2030	2.0854	0.036
250 m	4th	Average distance to bodies of Water/WingSpanmm	*r* = 0.1695	1.4793	0.053
500 m	5th	Number of trees/WingSpanmm	*r* = −0.2743	−2.9614	0.003
		Average distance to bodies of Water/WingSpanmm	*r* = 0.1978	2.0446	0.045
750 m	5th	Average distance to bodies of Water/WingSpanmm	*r* = 0.1881	1.9784	0.044
1000 m	5th	Total area of neighborhood Parks/WingSpanmm	*r* = −0.2368	−2.6796	0.007

## 5 Discussion

Our study revealed scale‐dependent relationships between environmental variables and butterfly diversity in Bogotá's urban parks. Taxonomic diversity showed stronger associations with local‐scale variables (250–500 m), while functional diversity responded more strongly to landscape heterogeneity at 750 m. The dbRDA analyses identified key predictors—average DBP and park area types—not detected by the other statistical methods. Consistent with our hypothesis that built‐up areas around parks would be associated with reduced species richness, we found that urbanization was indeed linked to lower butterfly taxonomic richness and diversity at the 250‐m scale. This suggests that the presence of built‐up areas negatively impacts microhabitat quality by limiting access to larval and adult food resources. As a result, the availability of diverse ecological niches decreases, leading to a reduction in the diversity of butterfly assemblages, even for common species. Urban environments, with their homogenized landscape, appear to intensify competition for limited resources, which ultimately reduces overall biodiversity (Lizee et al. 2016; Iserhard et al. 2019). These scale‐dependent effects have important management implications. At local scales (< 500 m), maintaining native plant richness and diverse green spaces is crucial for taxonomic diversity, while broader landscape strategies—including water body conservation and tree density at 750 m—are essential for functional diversity preservation. This highlights the need for multiscale conservation approaches in urban planning.

While our study provides valuable insights, several limitations must be acknowledged. The relatively small sample size (15 parks, 15 species, ~400 individuals) and focus on only two functional traits may limit generalizability. Future research should expand sampling efforts, include additional traits and life stages, and incorporate broader spatial contexts to comprehensively capture butterfly responses in urban environments. Despite these limitations, targeted management efforts focused on habitat restoration, microhabitat creation, and native species plantings are essential, particularly to support specialist species. Integrating these strategies into urban planning will help foster resilient, functionally diverse butterfly communities and sustain ecosystem services in tropical Andean cities like Bogotá.

### 5.1 Associations Between Urban Park Size, Local‐Scale Vegetation Structure, and Butterfly Assemblages

Medium and large parks exhibited the highest species richness, supporting six rare species (Tables S7 and S8), consistent with previous findings that larger urban areas support greater plant and floral resources (Nielsen et al. 2014; Pérez et al. 2019). While many species, such as the dominant species of Pieridae, prefer to fly over open areas (DeVries 1987), vegetation stratification can provide niches, shelter, and refuge for some cryptic species such as Hesperiidae species (Carneiro et al. 2014). Notably, native plant richness explained approximately 49% of the butterfly species diversity and dominance, revealing a stronger association than initially expected. It is important to mention that, out of the 144 plant species recorded in this study, 33 were native species (Table S13). These results are consistent with the findings made by other authors in cities such as Boston and New York (USA) and Beijing (China), where they found a positive relationship between the diversity of butterflies and the richness of native plant species and show a high abundance of flowers that provide nectar to the species at scales between local, 50 and 150 m around urban parks (Clark et al. 2007; Matteson and Langellotto 2010; Han et al. 2021). Similarly, some studies have found that higher native tree density is positively associated with increased larval density (Burghardt et al. 2009). Additionally, the distribution of native and exotic plants, along with the habitat complexity they create, is a critical factor affecting oviposition and the defoliation caused by caterpillars on their host plants (Clem and Held 2018).

Functional diversity indices were generally low across assemblages (Table S7), suggesting limited functional variation in urban butterfly communities. The dbRDA identified paved area and native species richness as important predictors at local scales, while pocket and neighborhood park areas influenced functional diversity at intermediate scales (250–750 m). Notably, FDis was the only functional metric explained at the local scale, with tree height accounting for 47.7% of its variation. This relationship suggests that taller trees create homogeneous canopies that favor generalist species while limiting niche availability for specialists, thereby reducing functional divergence (Aguirre‐Gutiérrez et al. 2017; Börschig et al. 2013). The low functional dispersion, richness, and divergence indicate reduced resilience to environmental disturbances and increased susceptibility to urban environmental filters such as exotic species, predation pressure, and vegetation changes (Laliberté and Legendre 2010).

Contrary to our hypothesis, the fourth‐corner analysis revealed that distance from water bodies showed significant positive associations with butterfly wing span at intermediate scales (500–750 m), with similar effect sizes (*r*≈0.19, *p* < 0.05 at both scales). While Kőrösi et al. (2022) identified landscape heterogeneity as a key driver of phenotypic variation and Guariento et al. (2023) emphasized soil nutrients in other ecosystems, our urban context revealed that distance from water bodies and tree height were positively associated with larger wing spans, while tree density showed negative associations. These patterns suggest that butterflies with larger wing spans, having greater dispersal capacity, can exploit habitats farther from water sources, while smaller‐winged species may be more dependent on the humid microclimates near water bodies.

### 5.2 Scale of Effect on Taxonomic and Functional Diversity of Butterfly Assemblages

Taxonomic diversity showed consistent associations with park configuration at multiple scales, with neighborhood parks (0.5–1.5 ha) emerging as excellent predictors despite not being part of Bogotá's main ecological structure (SDP 2020). At a 250 m radius, total neighborhood park area explained species richness (*q* = 0) and evenness (*q* = 1), while at 500 m it predicted assemblage structure, and at 1000 m it influenced dominance patterns (*q* > 2). Pocket parks complemented this effect by explaining taxonomic diversity at 500 m (Table 1). These findings align with studies showing that butterfly diversity responds to local‐scale resources within 250 m, where species actively move between habitats for resource acquisition (Flick et al. 2012; Clark et al. 2007). The consistent importance of these smaller parks suggests that they should be integrated into urban environmental planning for ecological connectivity.

Functional diversity metrics, particularly divergence and originality, responded to broader spatial scales (750 m), where water bodies significantly enhanced functional richness by providing unique microhabitats and essential resources for water absorption (Lin et al. 2023; Han et al. 2021). While our study captured important scale‐dependent patterns, the contrast between local‐scale butterfly movement (< 250 m) and potential landscape effects extending beyond 2 km in other systems (Bergman et al. 2018; Desaegher et al. 2022) suggests future research should examine scales > 1000 m. This is particularly relevant given the high species turnover among parks and the predominantly generalist assemblage (Table S8; Appendix S5), which may depend on complementary resources from peripheral ecosystems like the Eastern Hills and Bogotá River (sensu Ouin et al. 2004). Understanding these broader spatial associations, combined with host plant specialization dynamics (Gompert et al. 2015; Nylin et al. 2018), would provide comprehensive criteria for managing Bogotá's ecological structure.

### 5.3 Strategic Management and Conservation Approaches for Enhancing Butterfly Diversity in Bogotá's Urban and Periurban Landscapes

Hence, it becomes important to implement management strategies within the urban parks of Bogotá (in which there is homogeneity in their local variables) and protected natural areas such as the National Natural Park of Chingaza and the National Natural Park of Sumapáz, and other rural areas of the Sabana de Bogotá where butterfly species dispersal may occur. The potential that the city's green areas must function as connected corridors to each other and to natural and seminatural areas in larger geographic areas is highlighted. Furthermore, many small vegetation patches (given their local habitat variables) may play a crucial role in maintaining the regional pool of butterfly species (*sensu* Fahrig 2020). Bogotá, as in many other cities, has very little presence of native vegetation cover within the urban area, which is a common characteristic in cities that landscape, and the enrichment of urban areas has not been planned with ecological approaches that allow the establishment of strategies for the conservation of native flora and fauna. In this sense, future conservation strategies must adopt strategic planning models that account for both urban development and environmental conservation. Additionally, butterfly studies should consider all life stages, including eggs and larvae, and their relationships with host and food plants.

The results suggest that, if management and conservation strategies of urban parks in Bogotá are implemented within urban planning schemes, butterfly diversity in the city would be favored and ecosystem services supply could benefit Bogotá's citizens. According to our findings, we consider it necessary to manage structural heterogeneity and floristic diversity of Bogotá's urban parks to improve habitat quality for butterfly assemblages in the city. Additionally, since plants registered in this study are trees rather than herbaceous species, one simple strategy to maintain both the structure and composition of butterfly assemblages in Bogotá's urban parks is advocating for plant stratification and planting gardens with native plant species.

Urbanization's impact on biodiversity highlights the urgency of implementing habitat management strategies within urban environments, specifically focusing on enhancing the ecological integrity of urban green spaces by promoting functional diversity and ecological roles within butterfly assemblages. Our findings underscore the need for urban areas to adopt conservation‐oriented management strategies, recognizing the critical role of both natural and artificial green infrastructure in supporting urban biodiversity. In Bogotá, enhancing butterfly conservation through urban forestry in public green spaces is essential. Given that 77% of park plants are exotic species (Table S13), prioritizing native plants and tall trees in restoration efforts is crucial. Moreover, conservation actions should extend beyond a 750‐m radius around parks to create effective species corridors by increasing tree numbers. Furthermore, to safeguard specialist butterfly species with restricted distribution, targeted conservation efforts should focus on enhancing habitat quality within individual parks, especially those harboring rare species. Implementing site‐specific management practices, such as creating microhabitats, restoring native vegetation, and providing resources specific to these specialists (e.g., flowering plants, host plants, and water bodies), can help maintain their populations even in fragmented urban landscapes.

In conclusion, our findings highlight that Bogotá's urban parks, while housing a limited proportion of the region's butterfly species, play a crucial role in supporting common generalist species. The new species records for the city emphasize the potential for altitudinal shifts and the importance of ongoing monitoring in light of climate change (Table S8). To enhance butterfly diversity in the city, it is essential to implement management strategies that prioritize the structural heterogeneity and floristic diversity of urban parks. Promoting the use of native plants, advocating for plant stratification, and improving connectivity between urban green spaces and surrounding natural areas are key steps. Additionally, extending conservation efforts beyond park boundaries to create dispersal corridors will further bolster butterfly diversity. Future urban planning should incorporate these strategies to not only maintain but also enrich butterfly assemblages, ultimately benefiting both the city's biodiversity and its residents. The proposals for these conservation strategies can be found in Appendix S6.

## Author Contributions

**Nathali Coral‐Acosta:** conceptualization (equal), data curation (lead), formal analysis (equal), investigation (equal), visualization (equal), writing – original draft (lead), writing – review and editing (equal). **John Harold Castaño:** conceptualization (equal), investigation (equal), writing – review and editing (equal). **Darly Tatiana Rodríguez Jiménez:** data curation (supporting), formal analysis (equal), visualization (equal), writing – review and editing (supporting). **J. Nicolás Urbina‐Cardona:** conceptualization (equal), formal analysis (equal), investigation (equal), supervision (equal), visualization (equal), writing – review and editing (equal).

## Disclosure

The authors have nothing to report.

## Ethics Statement

The authors have nothing to report.

## Consent

If the work is accepted, the authors give their consent to publish this work in Urban Ecosystem.

## Conflicts of Interest

The authors declare no conflicts of interest.

## Supporting information


**Tables S1–S13:** ece372341‐sup‐0001‐TableS1‐S13.xlsx.

**Appendix S1‐S6:** ece372341‐sup‐0002‐AppendixS1‐S6.docx.

## Data Availability

Upon publication, the raw data will be available at “Scale‐Dependent Effects of Landscape Heterogeneity on Butterfly Functional and Taxonomic Diversity in Andean Urban Parks,” Mendeley Data, V1, doi: https://doi.org/10.17632/w8m7f98p34.1. https://data.mendeley.com/datasets/w8m7f98p34/1.
