# Supplementary material for: Scale‐Dependent Effects of Landscape Heterogeneity on Butterfly Functional and Taxonomic Diversity in Andean Urban Parks
Source: Ecol Evol. 2025 Oct 20;15(10):e72341. doi: 10.1002/ece3.72341 (PMC12537300; doi:10.1002/ece3.72341)
Supplement: Supplementary file 2 — Appendix S1‐S6: ece372341‐sup‐0002‐AppendixS1‐S6.docx. [file ECE3-15-e72341-s001.docx]

Supporting information:

*Ecology and Evolution*

**Scale-Dependent Effects of Landscape Composition on Butterfly Functional and Taxonomic Diversity in Andean Urban Parks**

**Appendix S1**

***Measurement of functional traits***

Each collected specimen was photographed dorsally and ventrally with a Canon EOS 60D digital camera with a Canon Φ58 mm 1:3.5-5.6 zoom lens and a Ring Flash Macro Led XT-103C at a standard distance of 15 cm. All images were edited using Adobe Photoshop CS6 Software (Figure S2).

To measure the traits concerning morphology, each of the images was calibrated in millimeters (Figure 1) using the distance and area tools of ImageJ Software Ver. 1.52ª (Rasband, 2018). For color traits, once the area of the fore and hind wings was selected, the "analyze RGB spectrum measurement" (red, green, and blue channels) tool was selected to obtain the total value for each of the channels of the color of dorsal and ventral sides of wings of individuals. These values were also obtained using ImageJ Ver. 1.52a software (Rasband, 2018). The RGB spectrum makes it possible to obtain the values of each channel so that taken as a whole, it represents a color (Moretti et al., 2017).


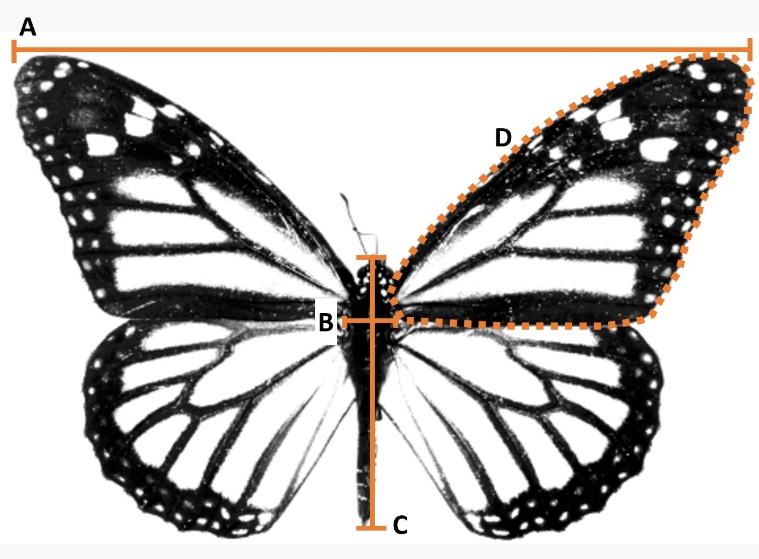


**Figure 1**. Measurement of morphological traits in relation to body size: A= wingspan, B= thorax width, C= body length, and D= area of the forewing and hindwing (e.g., dotted line on right forewing).

**Table 1**. Morphological functional traits measured in 268 individuals of the 15 butterfly species.

| **Name of Trait** | **Measurement** | **Unit** | **Description** | **Reference** |
| --- | --- | --- | --- | --- |
| Body length | Distance from head to pint of abdomen | mm | Strategy for obtaining resources and response of species to changes in environmental conditions | Aguirre-Gutiérres et al., (2017), Iserhard et al., (2019), Spaniol et al., (2019) |
| Wingspan | Distance between points of anterior wings extended | mm |  |  |
| Width of thorax | Distance between base of anterior wings | mm |  |  |
| Area of anterior right wing | Surface bounded by the contour of the wings | mm^2^ |  |  |
| Area of anterior left wing | Surface bounded by the contour of the wings | mm^2^ |  |  |
| Area of posterior right wing | Surface bounded by the contour of the wings | mm^2^ |  |  |
| Area of posterior left wing | Surface bounded by the contour of the wings | mm^2^ |  |  |
| Dorsal Total red (Red) Spectral Channel | Decimal value of the red channel of the forewings and hindwings on dorsal side | NA | Biotic and abiotic interactions effect pigmentation and influence the fitness of organisms | Zeuss et al., (2014), Ortiz-Acevedo et al., (2020) |
| Dorsal Total green (Green) Spectral Channel | Decimal value of the green channel of the forewings and hindwings on dorsal side | NA |  |  |
| Dorsal Total blue (Blue) Spectral Channel | Decimal value of the blue channel of the forewings and hindwings on dorsal side | NA |  |  |
| Dorsal Total red (Red) Spectral Channel | Decimal value of the red channel of the forewings and hindwings on ventral side | NA |  |  |
| Dorsal Total green (Green) Spectral Channel | Decimal value of the green channel of the forewings and hindwings on ventral side | NA |  |  |
| Dorsal Total blue (Blue) Spectral Channel | Decimal value of the blue channel of the forewings and hindwings on ventral side | NA |  |  |

**References**

Rasband, W. (2018). ImageJ 1.52a. National Institutes of Health, Bethesda, Maryland, USA. https://imagej.nih.gov/ij/, 1997-2018

Moretti, M., Dias, A. T., De Bello, F., Altermatt, F., Chown, S. L., Azcárate, F. M., Bell J. R., Fournier, B., Hedde, M., Hortal, J., Ibanez, S., Öckinger, E., Sousa, J. P., Ellers, J., & Berg, M. P. (2017). Handbook of protocols for standardized measurement of terrestrial invertebrate functional traits. Functional Ecology, 31 (3), 558-567. <https://doi.org/10.1111/1365-2435.12776>

**Appendix S2**

Linear regression adjustments between predictor variables for taxonomic and functional diversity indices across the five areas of influence ranging from local up to 1000 m around the focal patches.

**LOCAL SCALE**


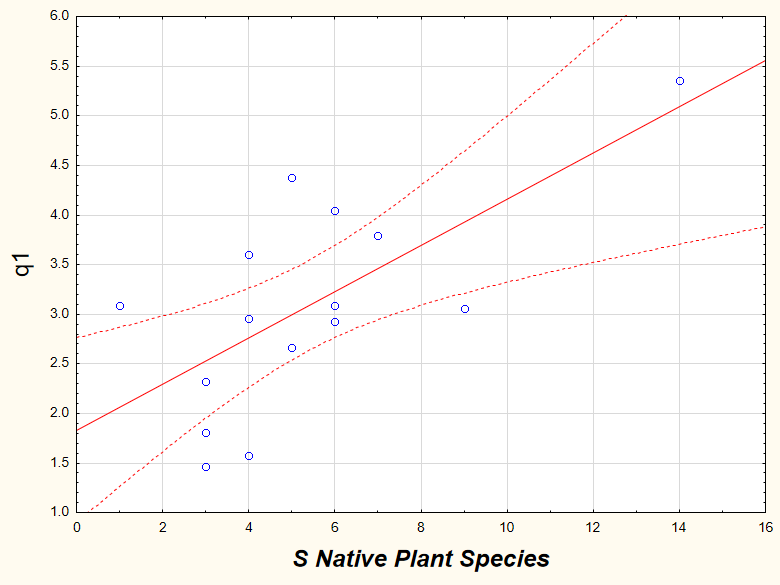


| **Park ID** | **q1** | ***S* Native Plant Species** |
| --- | --- | --- |
| P1 | 5.354 | 14 |
| P2 | 1.805 | 3 |
| P3 | 1.579 | 4 |
| P4 | 2.319 | 3 |
| P5 | 4.374 | 5 |
| P6 | 2.660 | 5 |
| P7 | 4.050 | 6 |
| P8 | 3.085 | 6 |
| P9 | 3.056 | 9 |
| P10 | 3.086 | 1 |
| P11 | 3.795 | 7 |
| P12 | 1.460 | 3 |
| P13 | 2.926 | 6 |
| P14 | 3.598 | 4 |
| P15 | 2.956 | 4 |

**Linear Regression**

Variable N R² Adj R² PMSE AIC BIC

q1 15 0.46 0.41 0.87 40.28 42.41

**Regression coefficients**

Coef Est. S.E. LL(95%) UL(95%) T p-value Mallows´Cp VIF

const 1.83 0.43 0.90 2.76 4.25 0.0010

S Native Plant Species 0.23 0.07 0.08 0.39 3.30 0.0058 10.89 1.00


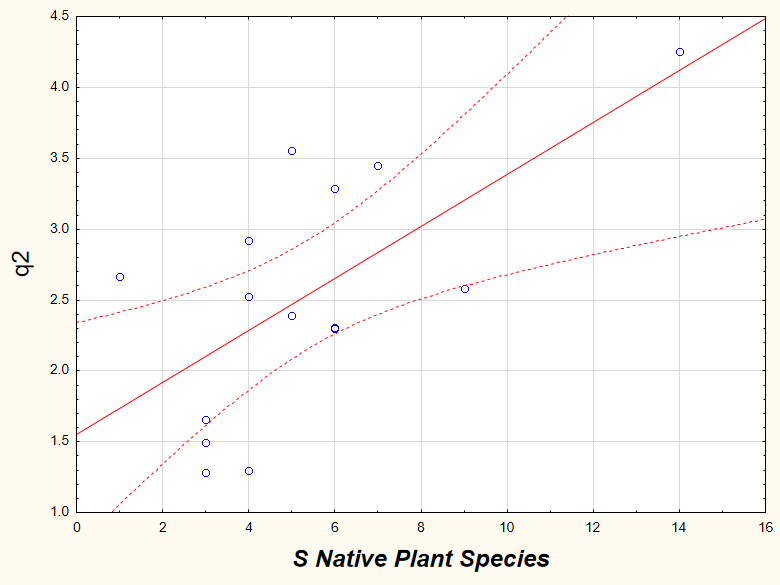


| **Park ID** | **q2** | ***S* Native Plant Species** |
| --- | --- | --- |
| P1 | 4.252 | 14 |
| P2 | 1.495 | 3 |
| P3 | 1.300 | 4 |
| P4 | 1.654 | 3 |
| P5 | 3.554 | 5 |
| P6 | 2.390 | 5 |
| P7 | 3.286 | 6 |
| P8 | 2.309 | 6 |
| P9 | 2.578 | 9 |
| P10 | 2.667 | 1 |
| P11 | 3.449 | 7 |
| P12 | 1.280 | 3 |
| P13 | 2.299 | 6 |
| P14 | 2.920 | 4 |
| P15 | 2.526 | 4 |

**Linear Regression**

Variable N R² Adj R² PMSE AIC BIC

q2 15 0.42 0.38 0.61 35.21 37.33

**Regression coefficients**

Coef Est. S.E. LL(95%) UL(95%) T p-value Mallows´Cp VIF

const 1.55 0.36 0.77 2.34 4.26 0.0009

S Native Plant Species 0.18 0.06 0.05 0.31 3.08 0.0088 9.46 1.00


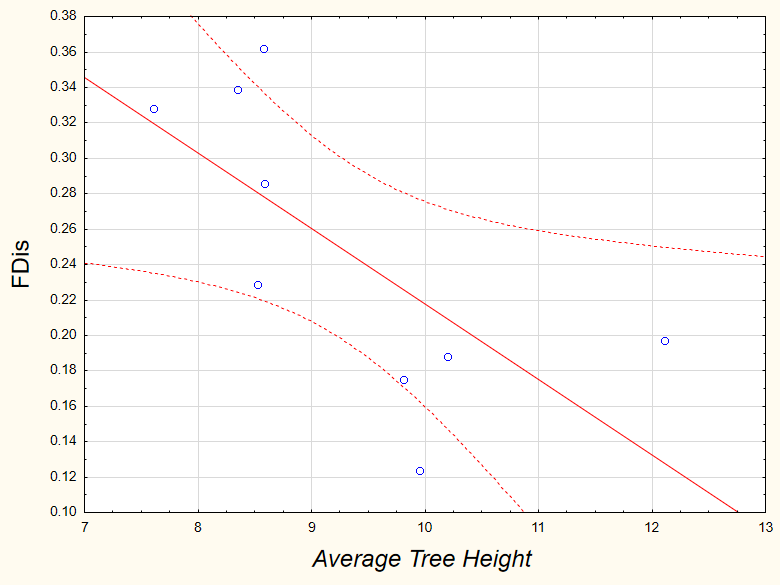


| **Park ID** | **FDis** | **Average Tree Height** |
| --- | --- | --- |
| P1 | 0.339 | 8.348 |
| P4 | 0.197 | 12.112 |
| P5 | 0.362 | 8.573 |
| P7 | 0.188 | 10.196 |
| P8 | 0.328 | 7.606 |
| P9 | 0.229 | 8.520 |
| P11 | 0.286 | 8.590 |
| P13 | 0.124 | 9.950 |
| P14 | 0.175 | 9.808 |

**Linear Regression**

Variable N R² Adj R² PMSE AIC BIC

FDis 9 0.48 0.40 0.01 -19.97 -19.38

**Regression coefficients**

Coef Est. S.E. LL(95%) UL(95%) T p-value Mallows´Cp VIF

const 0.64 0.16 0.27 1.02 4.08 0.0047

Average Tree Height -0.04 0.02 -0.08 -2.9E-03 -2.53 0.0390 6.42 1.00

**250 m SCALE**


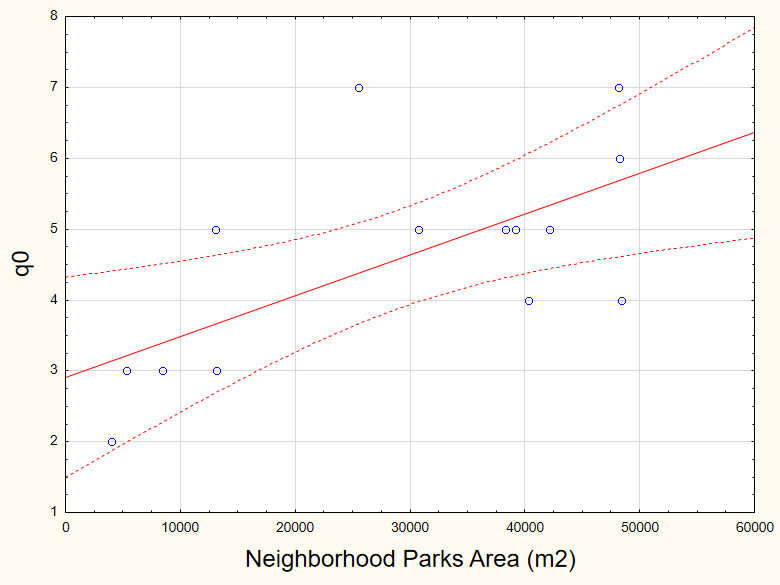


| **Park ID** | **q0** | **Neighborhood Parks Area (m2)** |
| --- | --- | --- |
| P1 | 7 | 25494.74 |
| P2 | 3 | 5328.16 |
| P3 | 3 | 8464.46 |
| P4 | 5 | 39211.14 |
| P5 | 7 | 48118.45 |
| P6 | 3 | 13134.81 |
| P7 | 6 | 48207.53 |
| P8 | 5 | 38304.68 |
| P9 | 5 |  |
| P10 | 4 | 48393.84 |
| P11 | 5 | 30759.69 |
| P12 | 2 | 4048.67 |
| P13 | 5 | 13061.25 |
| P14 | 5 | 42142.78 |
| P15 | 4 | 40314.88 |

**Linear Regression**

Variable N R² Adj R² PMSE AIC BIC

q0 14 0.42 0.37 1.89 48.54 50.46

**Regression coefficients**

Coef Est. S.E. LL(95%) UL(95%) T p-value Mallows´Cp VIF

const 2.91 0.65 1.49 4.32 4.48 0.0008

Neighborhood Parks Area (m.. 5.7E-05 2.0E-05 1.5E-05 1.0E-04 2.94 0.0124 8.64 1.00


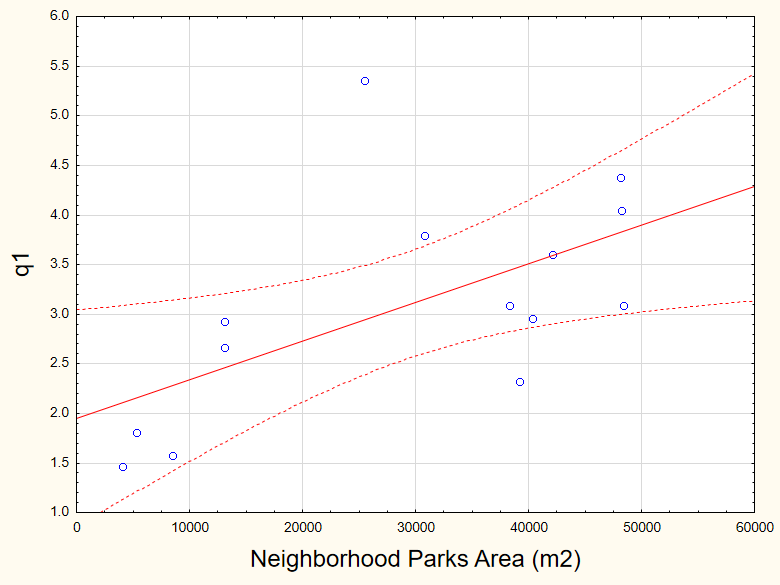


| **Park ID** | **q1** | **Neighborhood Parks Area (m2)** |
| --- | --- | --- |
| P1 | 5.354 | 25494.74 |
| P2 | 1.805 | 5328.16 |
| P3 | 1.579 | 8464.46 |
| P4 | 2.319 | 39211.14 |
| P5 | 4.374 | 48118.45 |
| P6 | 2.660 | 13134.81 |
| P7 | 4.050 | 48207.53 |
| P8 | 3.085 | 38304.68 |
| P9 | 3.056 |  |
| P10 | 3.086 | 48393.84 |
| P11 | 3.795 | 30759.69 |
| P12 | 1.460 | 4048.67 |
| P13 | 2.926 | 13061.25 |
| P14 | 3.598 | 42142.78 |
| P15 | 2.956 | 40314.88 |
|  |  |  |

**Linear Regression**

Variable N R² Adj R² PMSE AIC BIC

q1 14 0.36 0.30 1.07 41.33 43.25

**Regression coefficients**

Coef Est. S.E. LL(95%) UL(95%) T p-value Mallows´Cp VIF

const 1.95 0.50 0.86 3.04 3.88 0.0022

Neighborhood Parks Area (m.. 3.9E-05 1.5E-05 6.0E-06 7.2E-05 2.57 0.0244 6.62 1.00


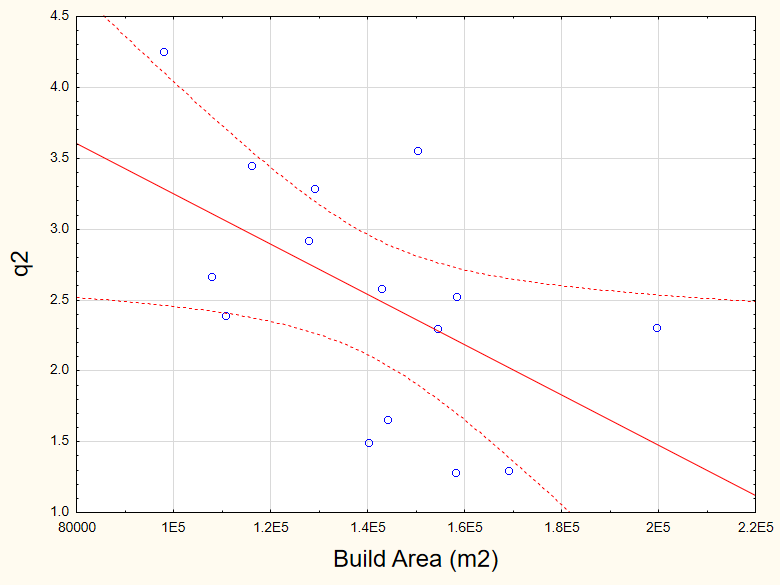


| **Park ID** | **q2** | **Build Area (m2)** |
| --- | --- | --- |
| P1 | 4.252 | 97992.70 |
| P2 | 1.495 | 140115.04 |
| P3 | 1.300 | 169017.84 |
| P4 | 1.654 | 144128.75 |
| P5 | 3.554 | 150278.30 |
| P6 | 2.390 | 110724.68 |
| P7 | 3.286 | 129091.73 |
| P8 | 2.309 | 199660.49 |
| P9 | 2.578 | 142870.53 |
| P10 | 2.667 | 107929.90 |
| P11 | 3.449 | 116082.64 |
| P12 | 1.280 | 158042.31 |
| P13 | 2.299 | 154386.27 |
| P14 | 2.920 | 127914.40 |
| P15 | 2.526 | 158302.00 |

**Linear Regression**

Variable N R² Adj R² PMSE AIC BIC

q2 15 0.29 0.24 0.84 38.22 40.34

**Regression coefficients**

Coef Est. S.E. LL(95%) UL(95%) T p-value Mallows´Cp VIF

const 5.02 1.09 2.66 7.38 4.60 0.0005

Build Area (m2) -1.8E-05 7.7E-06 -3.4E-05 -1.2E-06 -2.32 0.0372 5.38 1.00

**500 m SCALE**


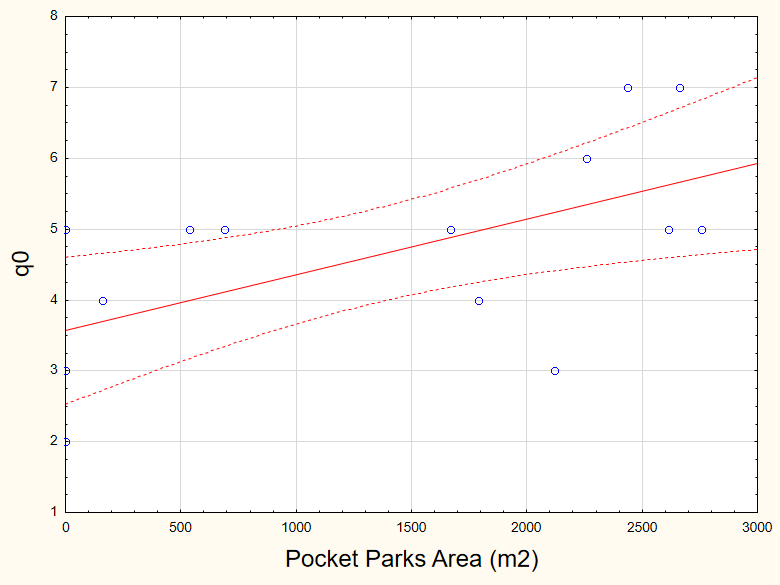


| **Park ID** | **q0** | **Pocket Parks Area (m2)** |
| --- | --- | --- |
| P1 | 7 | 2436.833 |
| P2 | 3 | 2118.135 |
| P3 | 3 |  |
| P4 | 5 | 690.190 |
| P5 | 7 | 2663.395 |
| P6 | 3 |  |
| P7 | 6 | 2260.207 |
| P8 | 5 | 1670.499 |
| P9 | 5 | 537.024 |
| P10 | 4 | 1791.581 |
| P11 | 5 | 2757.015 |
| P12 | 2 |  |
| P13 | 5 |  |
| P14 | 5 | 2615.764 |
| P15 | 4 | 159.247 |

**Linear Regression**

Variable N R² Adj R² PMSE AIC BIC

q0 11 0.16 0.06 1.88 38.68 39.88

**Regression coefficients**

Coef Est. S.E. LL(95%) UL(95%) T p-value Mallows´Cp VIF

const 4.16 0.81 2.33 5.98 5.16 0.0006

Pocket Parks Area (m2) 5.2E-04 4.0E-04 -3.9E-04 1.4E-03 1.30 0.2275 1.68 1.00


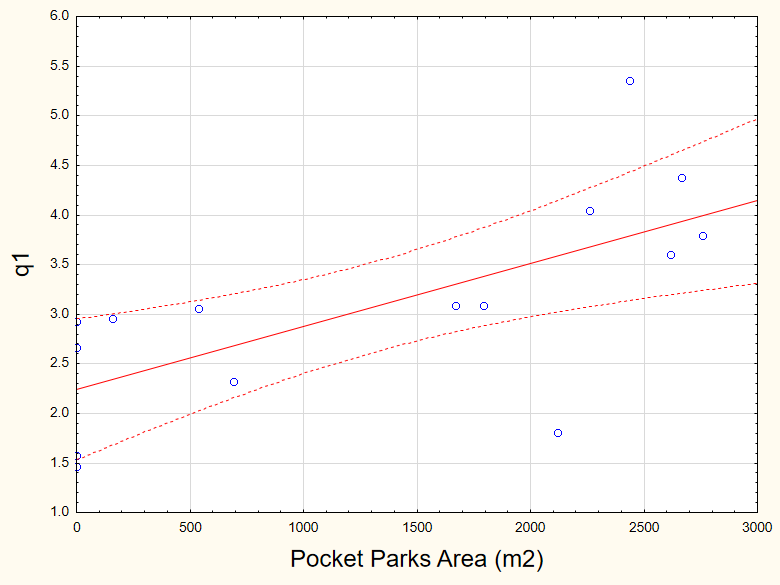


| **Park ID** | **q1** | **Poket Parks Area (m2)** |
| --- | --- | --- |
| P1 | 5.354 | 2436.833 |
| P2 | 1.805 | 2118.135 |
| P3 | 1.579 |  |
| P4 | 2.319 | 690.190 |
| P5 | 4.374 | 2663.395 |
| P6 | 2.660 |  |
| P7 | 4.050 | 2260.207 |
| P8 | 3.085 | 1670.499 |
| P9 | 3.056 | 537.024 |
| P10 | 3.086 | 1791.581 |
| P11 | 3.795 | 2757.015 |
| P12 | 1.460 |  |
| P13 | 2.926 |  |
| P14 | 3.598 | 2615.764 |
| P15 | 2.956 | 159.247 |

**Linear Regression**

Variable N R² Adj R² PMSE AIC BIC

q1 11 0.28 0.20 1.05 32.08 33.28

**Regression coefficients**

Coef Est. S.E. LL(95%) UL(95%) T p-value Mallows´Cp VIF

const 2.40 0.60 1.05 3.75 4.02 0.0030

Poket Parks Area (m2) 5.6E-04 3.0E-04 -1.1E-04 1.2E-03 1.88 0.0927 3.54 1.00


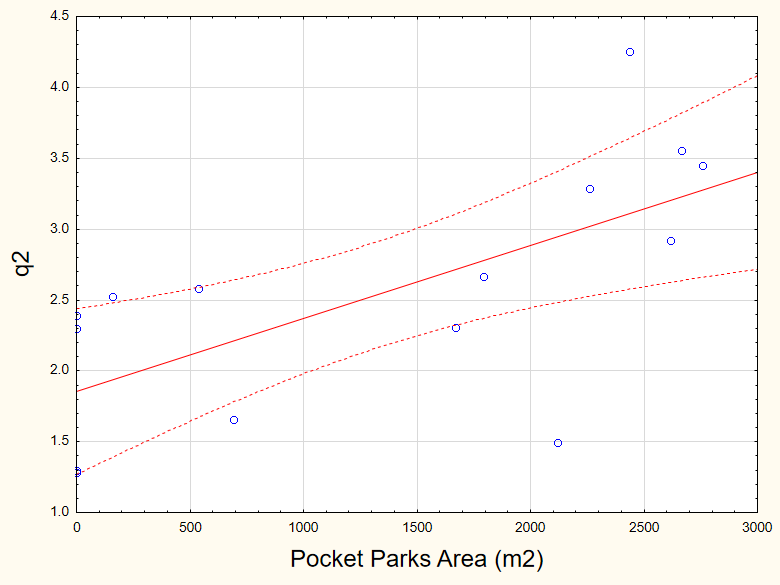


| **Park ID** | **q2** | **Poket Parks Area (m2)** |
| --- | --- | --- |
| P1 | 4.252 | 2436.833 |
| P2 | 1.495 | 2118.135 |
| P3 | 1.300 |  |
| P4 | 1.654 | 690.190 |
| P5 | 3.554 | 2663.395 |
| P6 | 2.390 |  |
| P7 | 3.286 | 2260.207 |
| P8 | 2.309 | 1670.499 |
| P9 | 2.578 | 537.024 |
| P10 | 2.667 | 1791.581 |
| P11 | 3.449 | 2757.015 |
| P12 | 1.280 |  |
| P13 | 2.299 |  |
| P14 | 2.920 | 2615.764 |
| P15 | 2.526 | 159.247 |

**Linear Regression**

Variable N R² Adj R² PMSE AIC BIC

q2 11 0.30 0.22 0.75 27.91 29.11

**Regression coefficients**

Coef Est. S.E. LL(95%) UL(95%) T p-value Mallows´Cp VIF

const 1.92 0.49 0.80 3.04 3.89 0.0037

Poket Parks Area (m2) 4.9E-04 2.5E-04 -7.4E-05 1.0E-03 1.96 0.0810 3.86 1.00


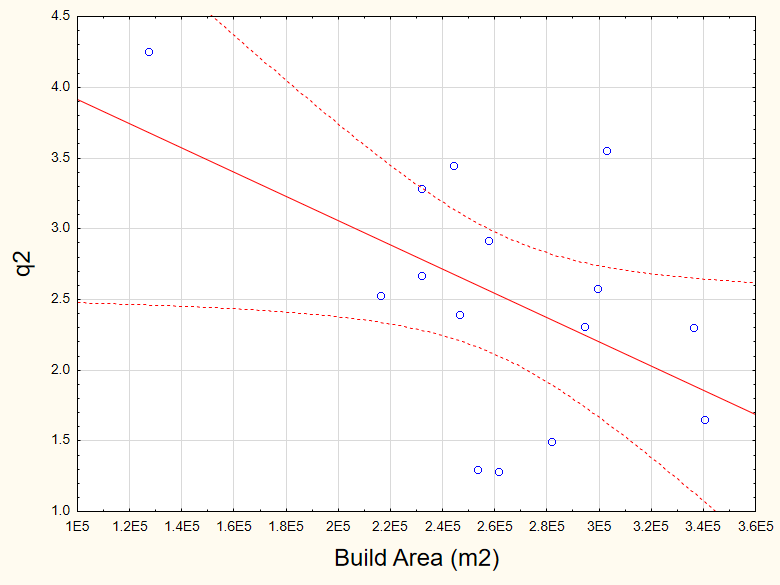


| **Park ID** | **q2** | **Build Area (m2)** |
| --- | --- | --- |
| P1 | 4.252 | 127276.50 |
| P2 | 1.495 | 281623.41 |
| P3 | 1.300 | 253501.63 |
| P4 | 1.654 | 340398.85 |
| P5 | 3.554 | 303038.13 |
| P6 | 2.390 | 246378.78 |
| P7 | 3.286 | 231752.17 |
| P8 | 2.309 | 294273.11 |
| P9 | 2.578 | 299379.29 |
| P10 | 2.667 | 232095.86 |
| P11 | 3.449 | 244353.14 |
| P12 | 1.280 | 261312.86 |
| P13 | 2.299 | 336391.70 |
| P14 | 2.920 | 257577.61 |
| P15 | 2.526 | 216301.17 |

**Linear Regression**

Variable N R² Adj R² PMSE AIC BIC

q2 15 0.27 0.21 0.80 38.74 40.87

**Regression coefficients**

Coef Est. S.E. LL(95%) UL(95%) T p-value Mallows´Cp VIF

const 4.77 1.05 2.51 7.03 4.56 0.0005

Build Area (m2) -8.6E-06 3.9E-06 -1.7E-05 -7.4E-08 -2.18 0.0483 4.75 1.00

**750 m SCALE**


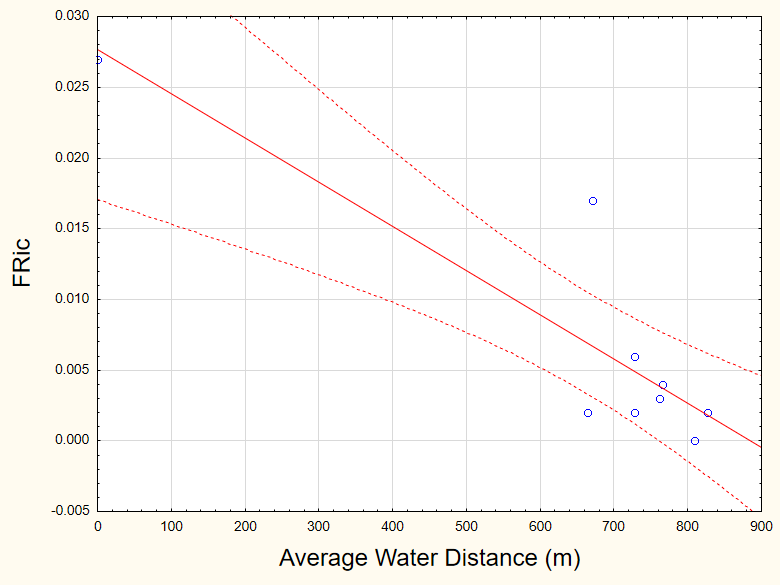


| **Park ID** | **FRic** | **Average Water Distance (m)** |
| --- | --- | --- |
| P1 | 0.027 |  |
| P4 | 0.002 | 663.545 |
| P5 | 0.017 | 670.28 |
| P7 | 0.003 | 762.104 |
| P8 | 0.006 | 728.38 |
| P9 | 0.002 | 826.694 |
| P11 | 0.004 | 766.192 |
| P13 | 0.000 | 809.311 |
| P14 | 0.002 | 728.067 |

**Linear Regression**

Variable N R² Adj R² PMSE AIC BIC

FRic 8 0.32 0.20 5.4E-05 -59.13 -58.89

**Regression coefficients**

Coef Est. S.E. LL(95%) UL(95%) T p-value Mallows´Cp VIF

const 0.04 0.02 -0.01 0.10 1.86 0.1117

Average Water Distance (m).. -5.1E-05 3.1E-05 -1.3E-04 2.4E-05 -1.67 0.1459 2.79 1.00


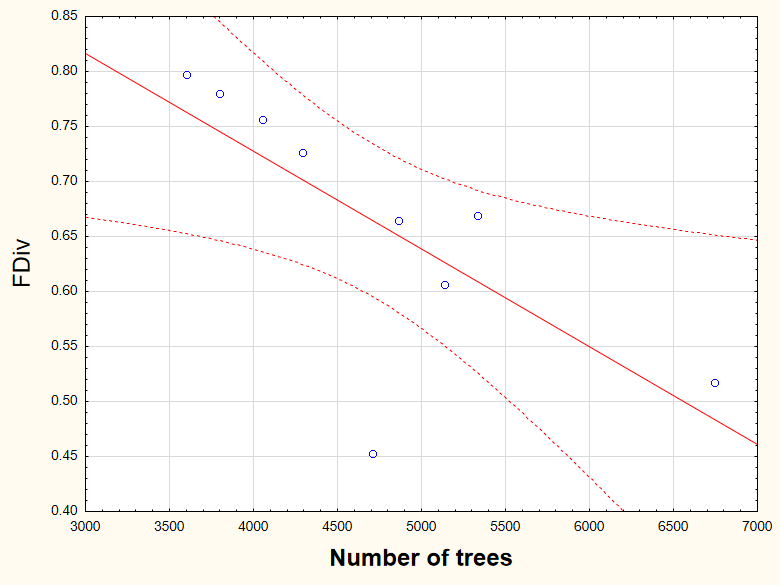


| **Park ID** | **FDiv** | **Number of trees** |
| --- | --- | --- |
| P1 | 0.669 | 5335 |
| P4 | 0.726 | 4293 |
| P5 | 0.756 | 4051 |
| P7 | 0.606 | 5136 |
| P8 | 0.780 | 3798 |
| P9 | 0.797 | 3604 |
| P11 | 0.665 | 4866 |
| P13 | 0.453 | 4706 |
| P14 | 0.517 | 6747 |

**Linear Regression**

Variable N R² Adj R² PMSE AIC BIC

FDiv 9 0.52 0.45 0.01 -14.46 -13.86

**Regression coefficients**

Coef Est. S.E. LL(95%) UL(95%) T p-value Mallows´Cp VIF

const 1.08 0.16 0.72 1.45 6.96 0.0002

Number of trees -8.9E-05 3.2E-05 -1.7E-04 -1.2E-05 -2.75 0.0285 7.57 1.00


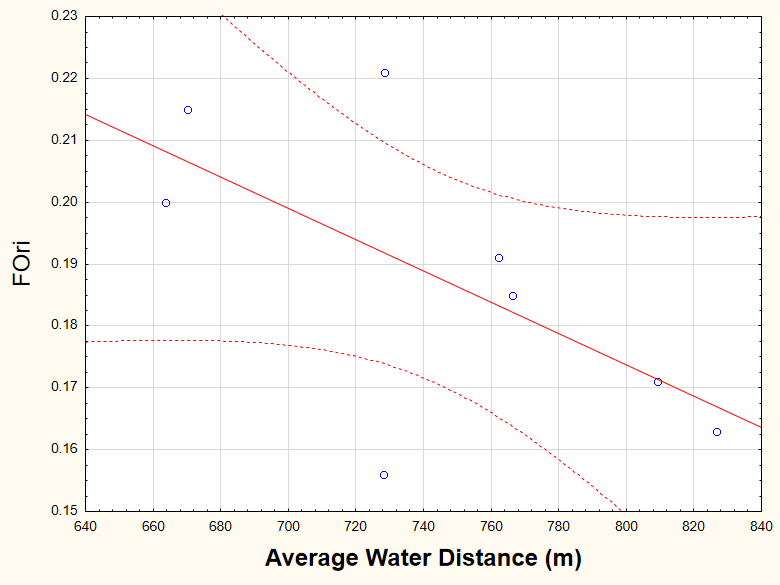


| **Park ID** | **FOri** | **Average Water Distance (m)** |
| --- | --- | --- |
| P1 | 0.235 |  |
| P4 | 0.200 | 663.545 |
| P5 | 0.215 | 670.28 |
| P7 | 0.191 | 762.104 |
| P8 | 0.221 | 728.38 |
| P9 | 0.163 | 826.694 |
| P11 | 0.185 | 766.192 |
| P13 | 0.171 | 809.311 |
| P14 | 0.156 | 728.067 |

**Linear Regression**

Variable N R² Adj R² PMSE AIC BIC

FOri 8 0.40 0.30 5.6E-04 -36.32 -36.08

**Regression coefficients**

Coef Est. S.E. LL(95%) UL(95%) T p-value Mallows´Cp VIF

const 0.38 0.09 0.14 0.61 3.96 0.0075

Average Water Distance (m).. -2.5E-04 1.3E-04 -5.6E-04 5.8E-05 -1.99 0.0941 3.95 1.00

**1000 m SCALE**


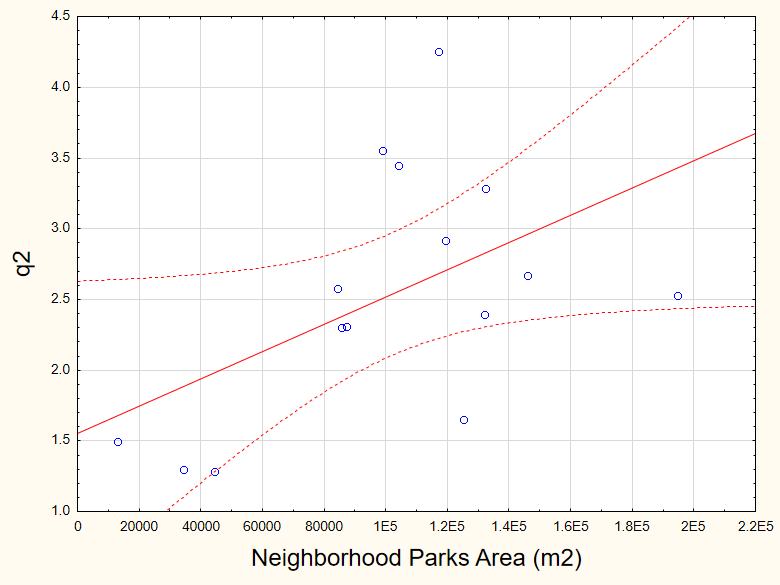


| **Park ID** | **q2** | **Neighborhood Parks Area (m2)** |
| --- | --- | --- |
| P1 | 4.252 | 117002.29 |
| P2 | 1.495 | 13090.60 |
| P3 | 1.300 | 34500.05 |
| P4 | 1.654 | 125395.81 |
| P5 | 3.554 | 99068.70 |
| P6 | 2.390 | 131963.25 |
| P7 | 3.286 | 132392.50 |
| P8 | 2.309 | 87136.56 |
| P9 | 2.578 | 84503.63 |
| P10 | 2.667 | 146125.69 |
| P11 | 3.449 | 104282.20 |
| P12 | 1.280 | 44409.75 |
| P13 | 2.299 | 85635.60 |
| P14 | 2.920 | 119361.48 |
| P15 | 2.526 | 194715.36 |

**Linear Regression**

Variable N R² Adj R² PMSE AIC BIC

q2 15 0.26 0.20 0.81 38.87 40.99

**Regression coefficients**

Coef Est. S.E. LL(95%) UL(95%) T p-value Mallows´Cp VIF

const 1.56 0.50 0.48 2.63 3.13 0.0080

Neighborhood Parks Area (m.. 9.6E-06 4.5E-06 -6.9E-08 1.9E-05 2.14 0.0514 4.60 1.00


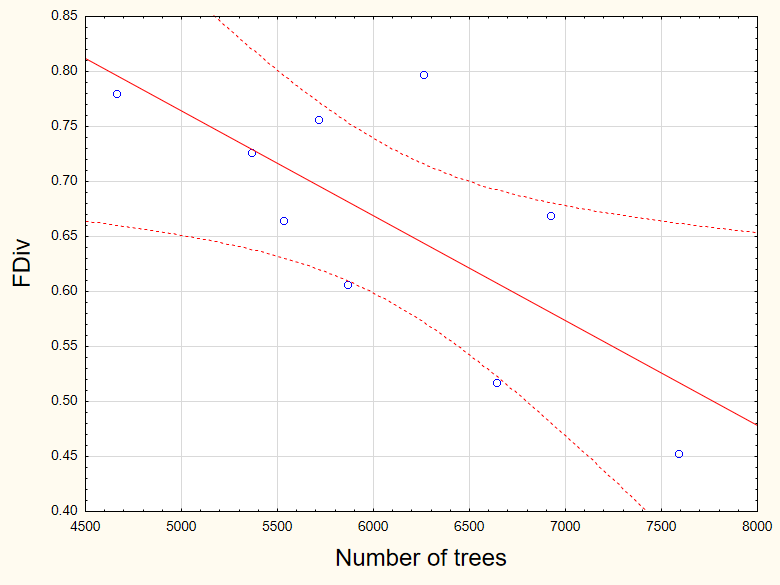


| **Park ID** | **FDiv** | **Number of trees** |
| --- | --- | --- |
| P1 | 0.669 | 6920 |
| P4 | 0.726 | 5364 |
| P5 | 0.756 | 5712 |
| P7 | 0.606 | 5866 |
| P8 | 0.780 | 4663 |
| P9 | 0.797 | 6259 |
| P11 | 0.665 | 5531 |
| P13 | 0.453 | 7586 |
| P14 | 0.517 | 6643 |

**Linear Regression**

Variable N R² Adj R² PMSE AIC BIC

FDiv 9 0.51 0.44 0.01 -14.28 -13.68

**Regression coefficients**

Coef Est. S.E. LL(95%) UL(95%) T p-value Mallows´Cp VIF

const 1.24 0.22 0.73 1.75 5.74 0.0007

Number of trees -9.5E-05 3.5E-05 -1.8E-04 -1.2E-05 -2.70 0.0307 7.28 1.00

**Appendix S3**

Distance based redundancy analysis (dbRDA) of the multivariate response variables such as assemblage structure (using the Bray-Curtis similarity index) and overall functional diversity (assessed as the Euclidean distance between sites based on the six functional diversity indices) across the five areas of influence ranging from local up to 1000 m around the focal patches).


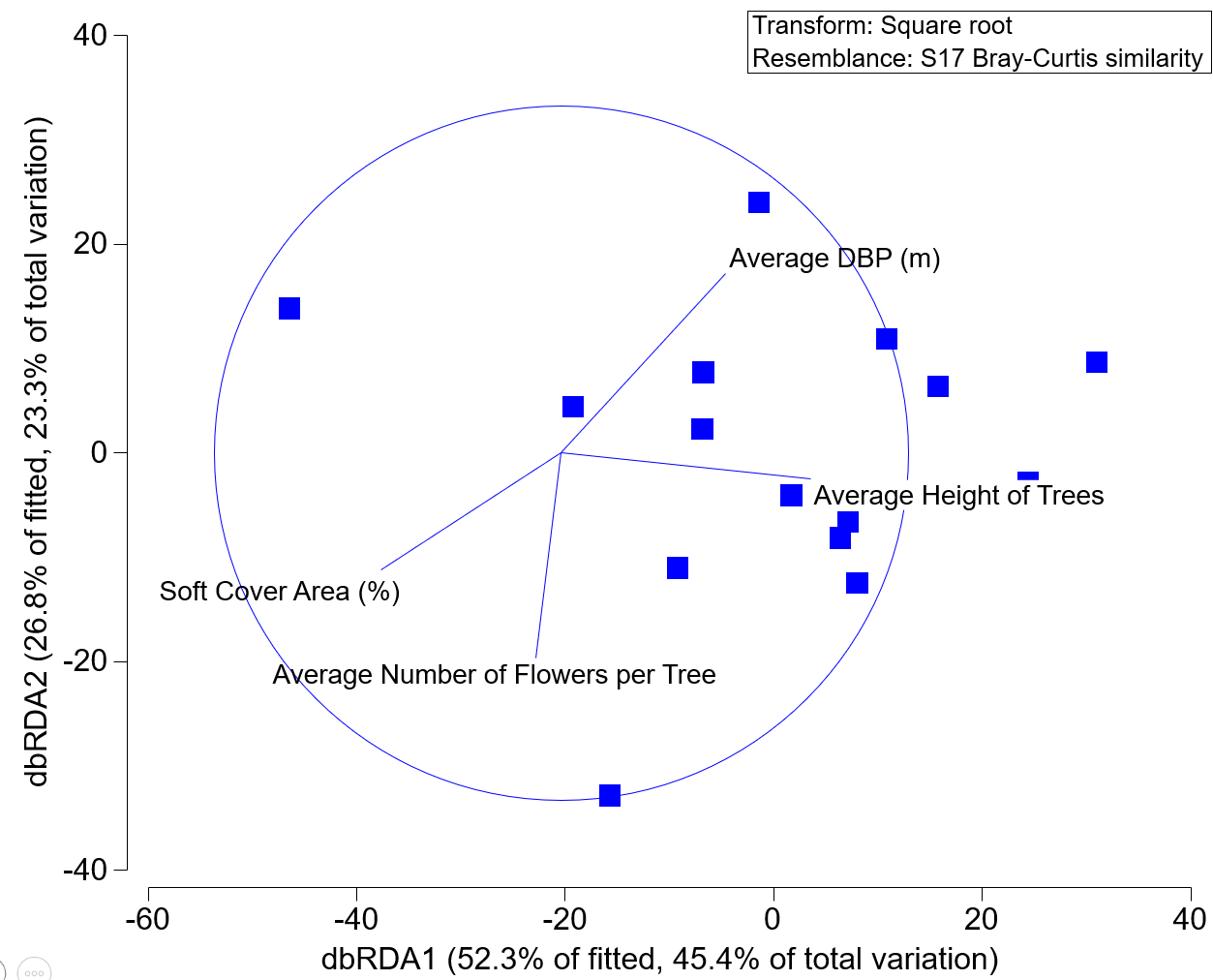


Bray-Curtis similarity explained at local scale


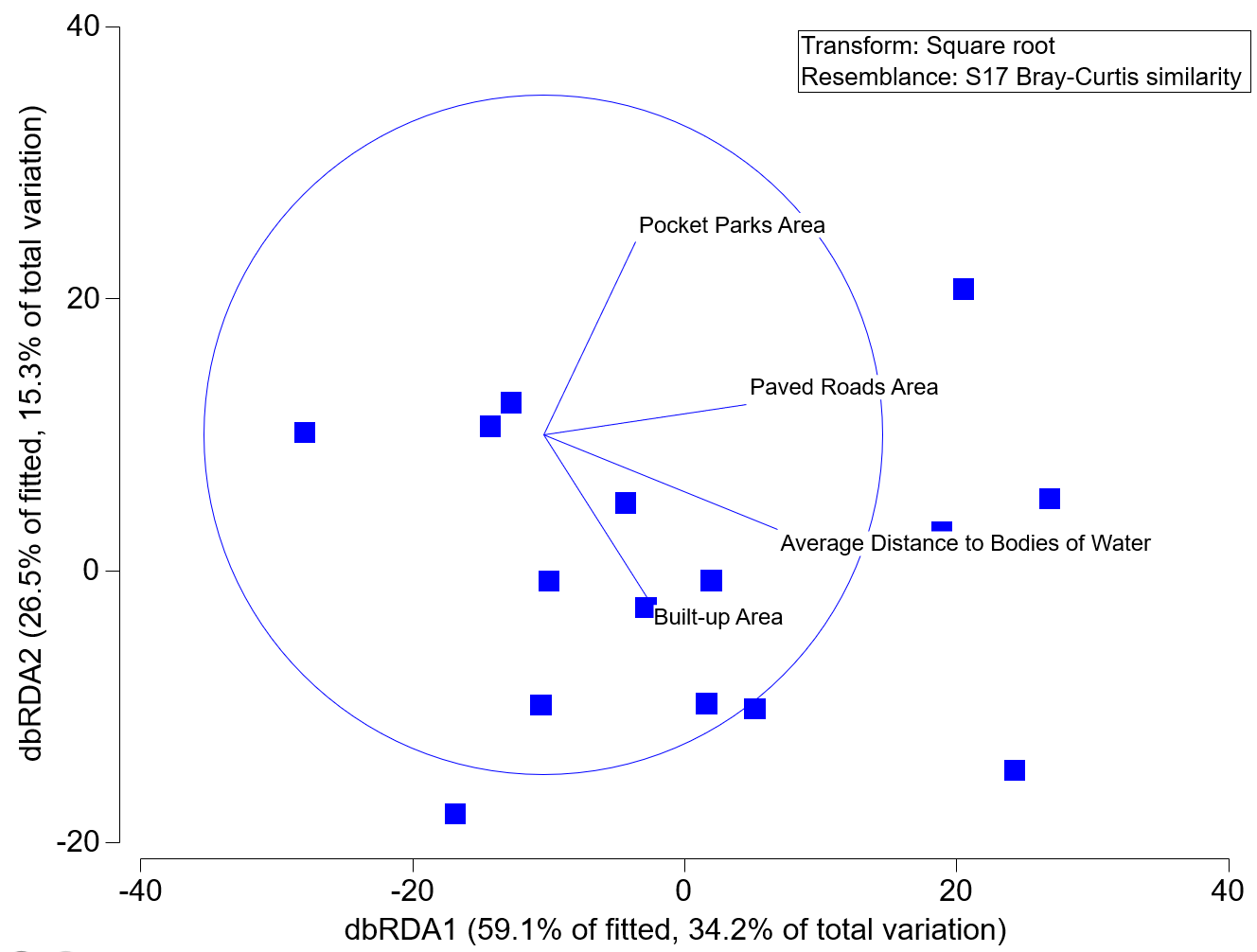


Bray-Curtis similarity explained at 250m scale


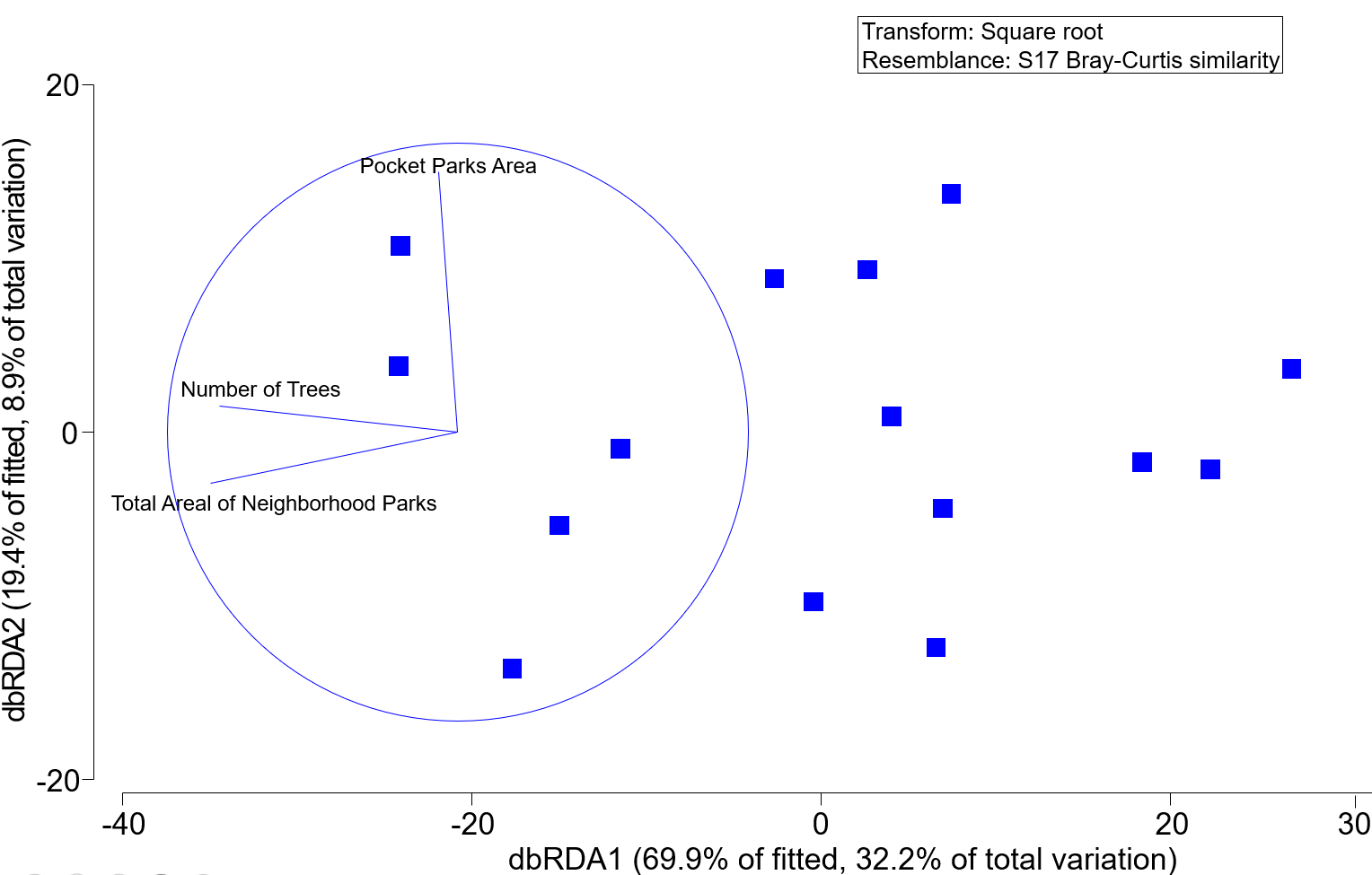


Bray-Curtis similarity explained at 500m scale


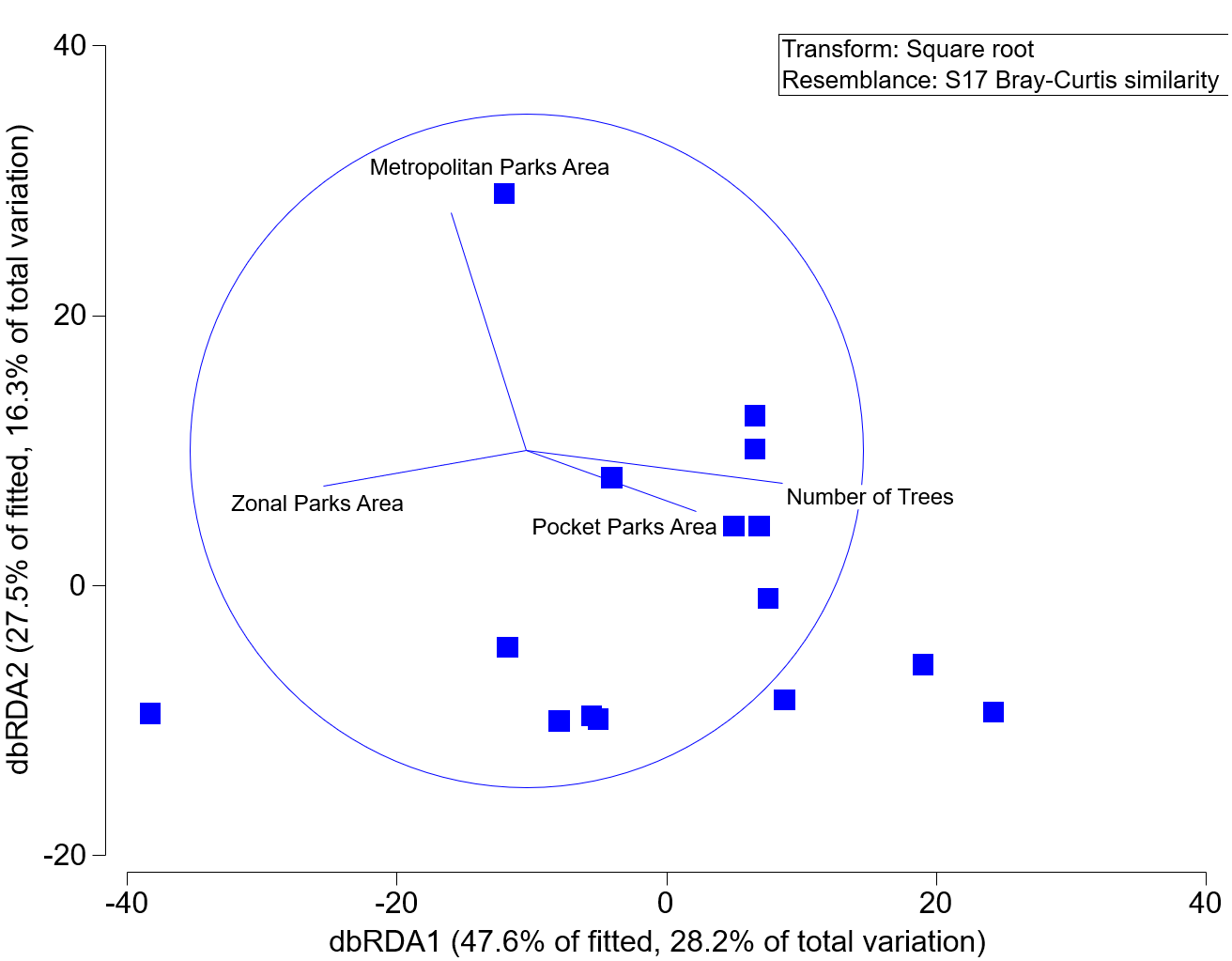


Bray-Curtis similarity explained at 750m scale


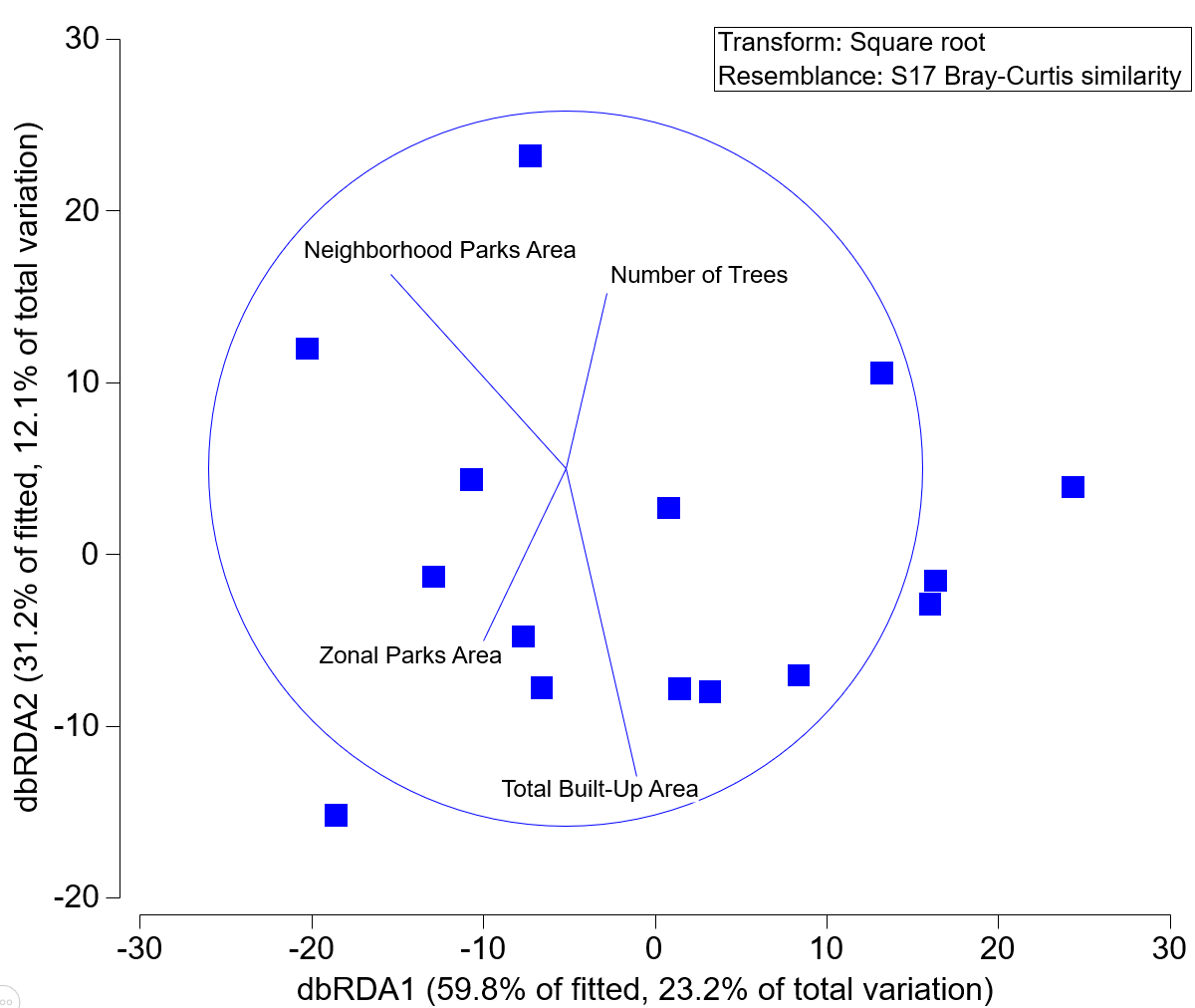


Bray-Curtis similarity explained at 1000m scale


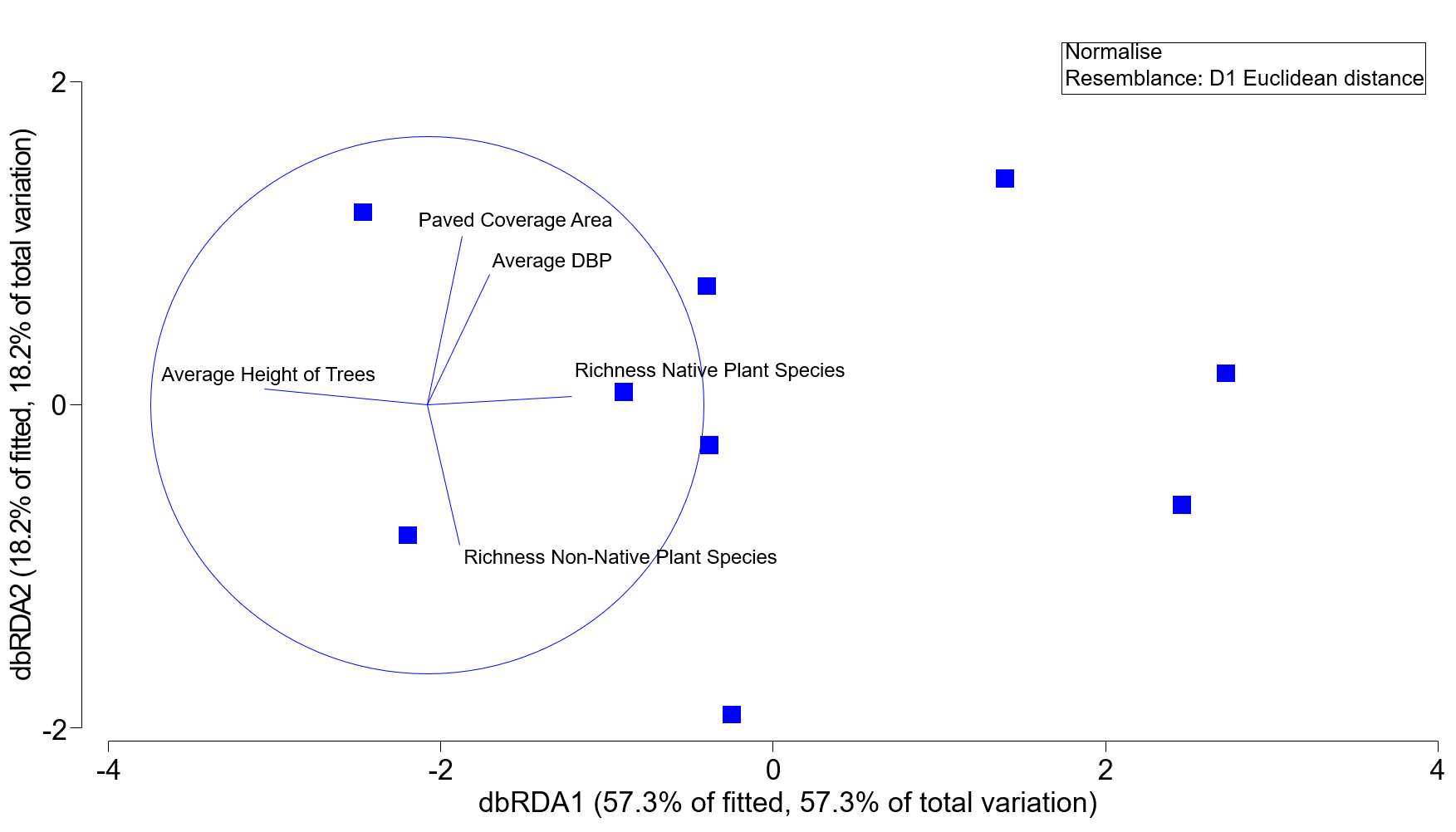


Overall functional diversity explained at local scale


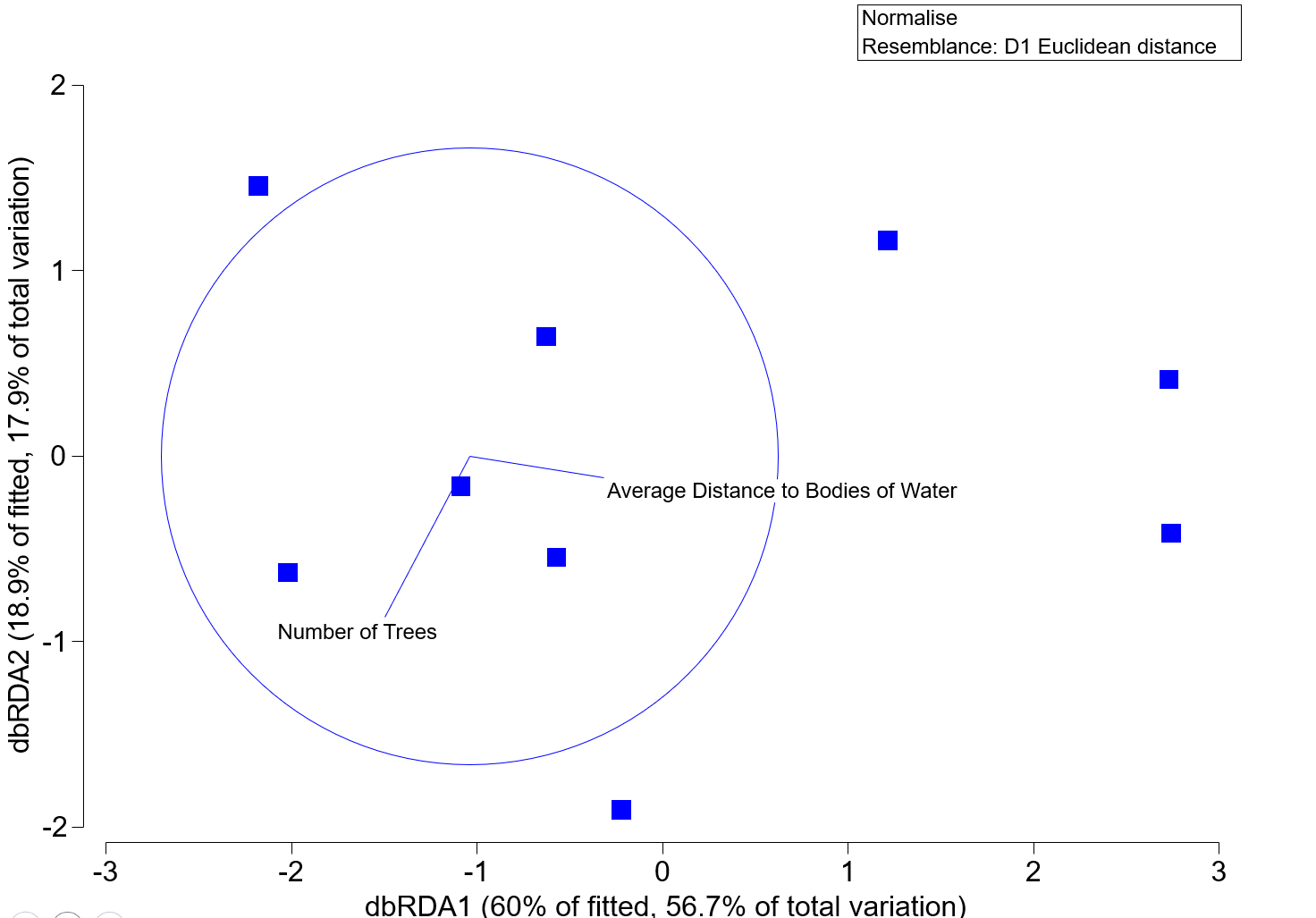


Overall functional diversity explained at 250m scale


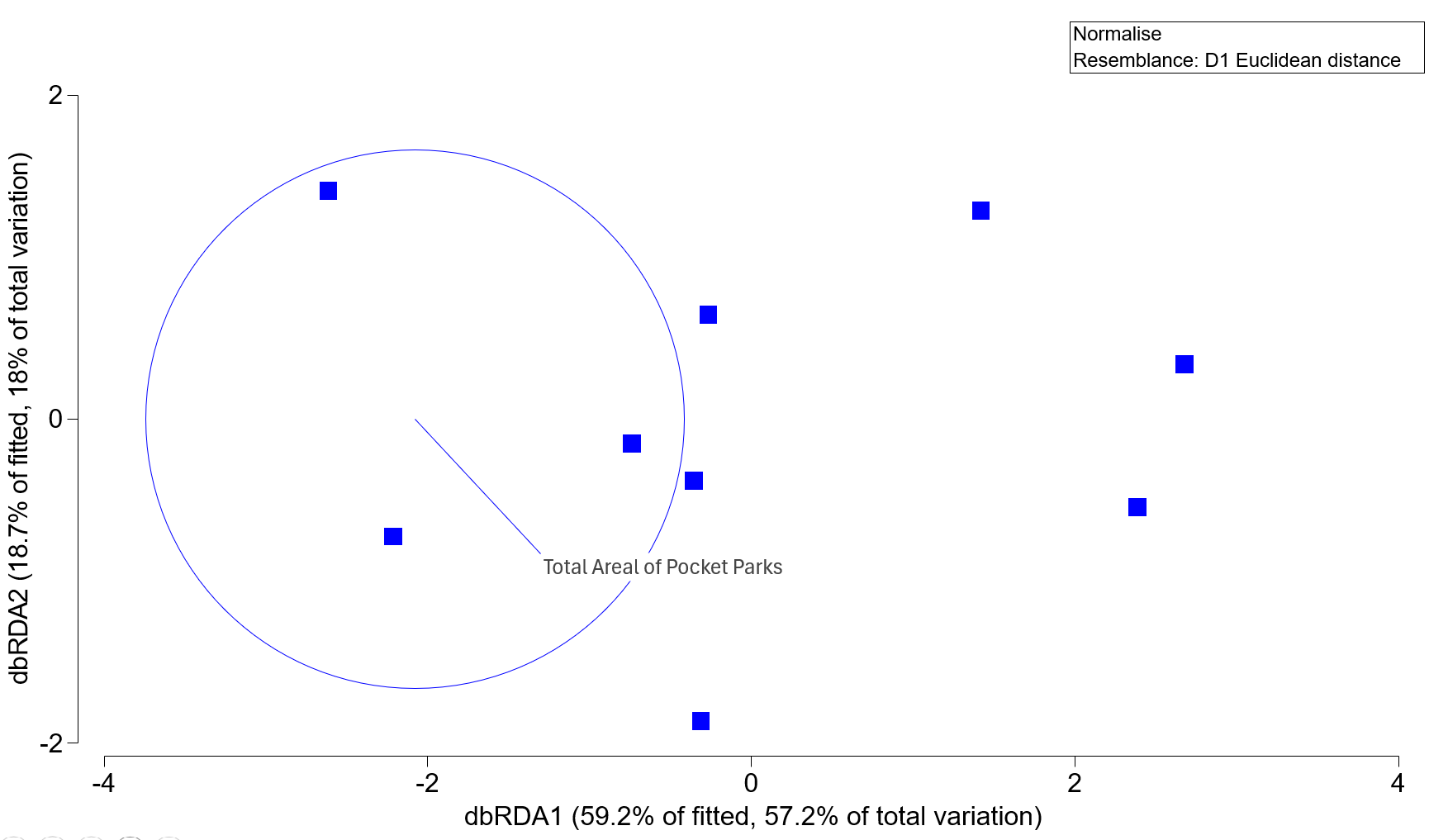


Overall functional diversity explained at 500m scale


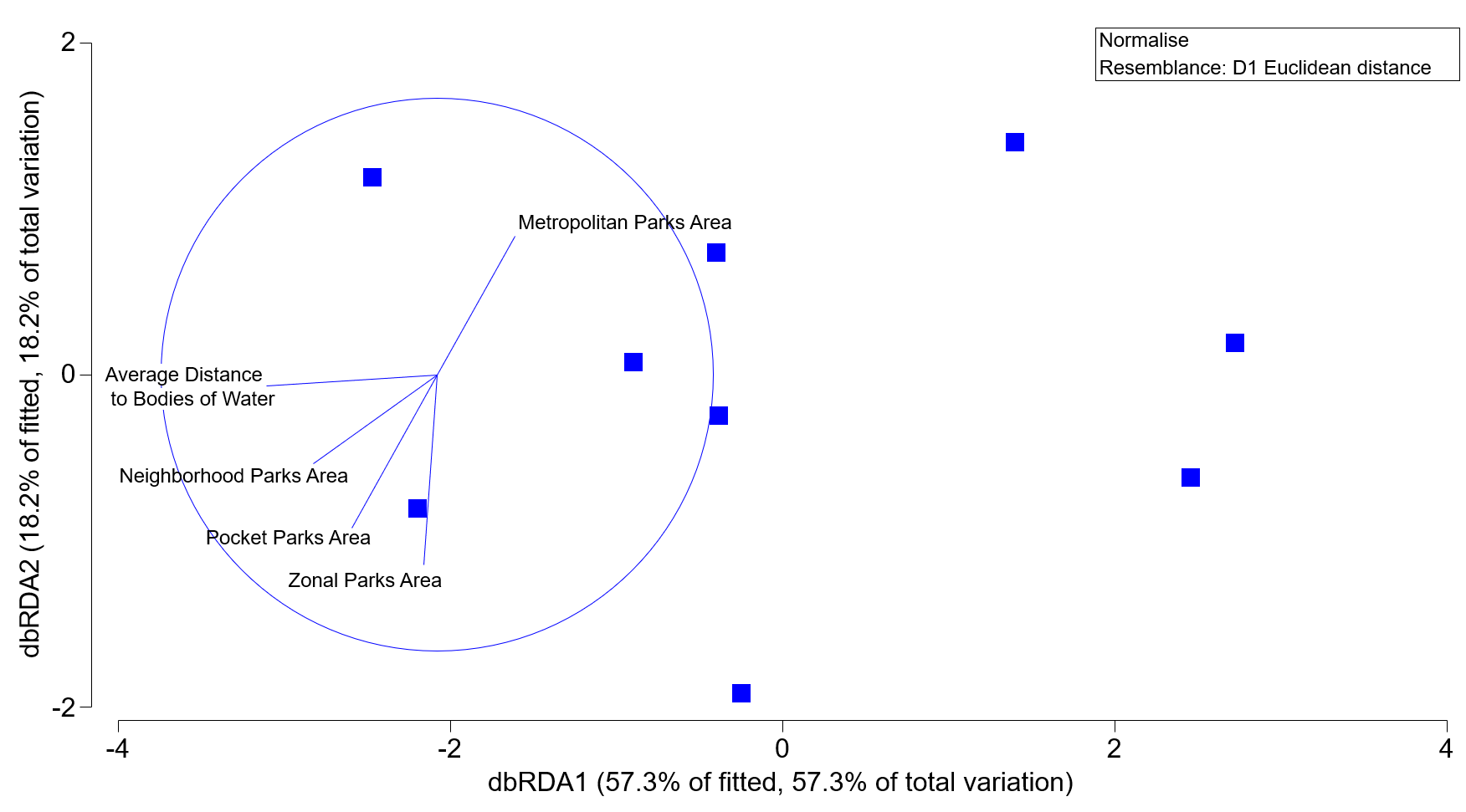


Overall functional diversity explained at 750m scale


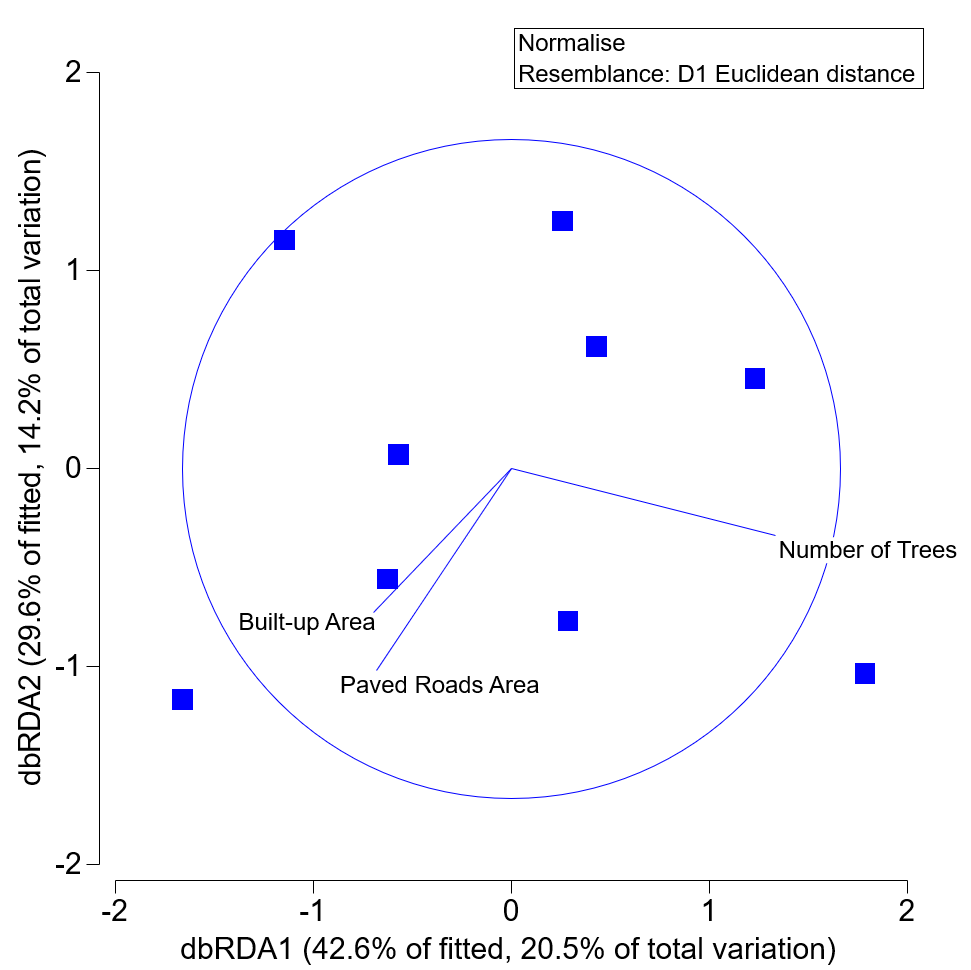


Overall functional diversity explained at 1000m scale

**Appendix S4**

Results of RLQ Analysis Across Five Spatial Scales (Local, 250m, 500m, 750m, and 1000m Around the Focal Sampling Patch.


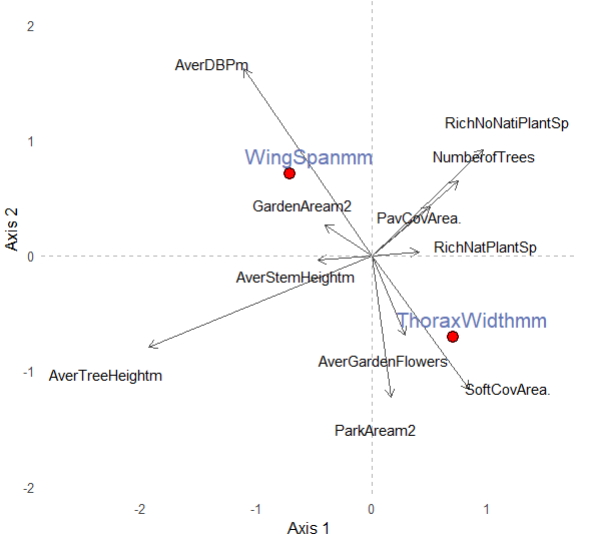

RLQ local scale


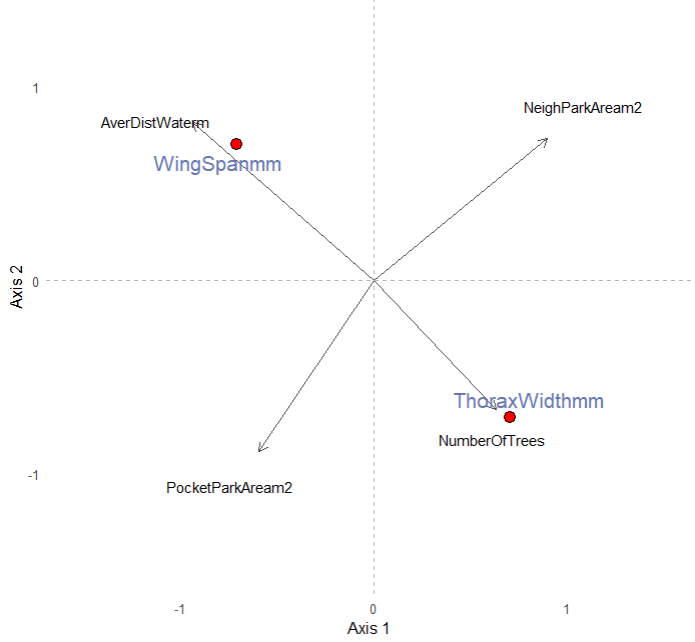

RLQ 250 m scale


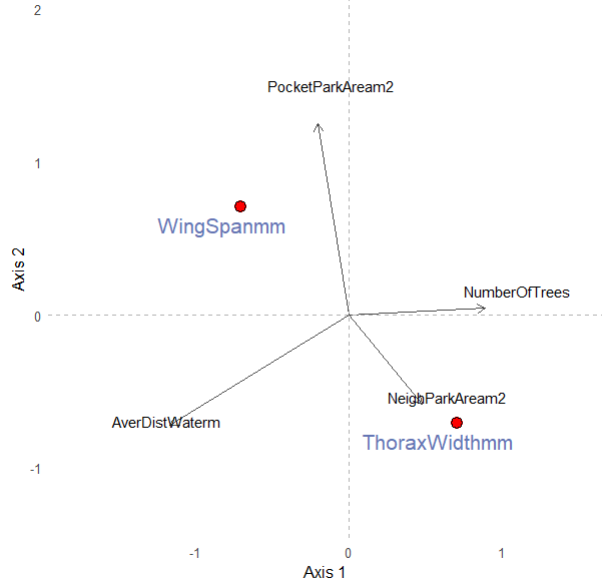


RLQ 500 m scale


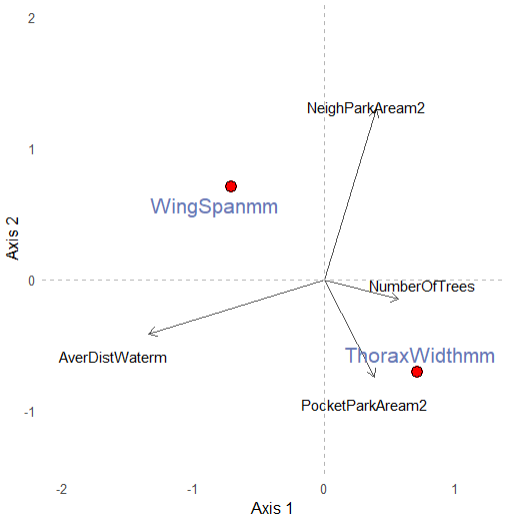

RLQ 750 m scale


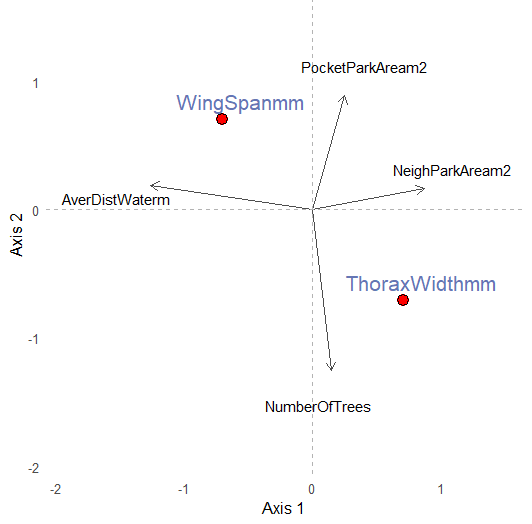


RLQ 1000 m scale

**Appendix S5**

***Butterfly assemblage composition in urban parks in Bogotá***

Considering previous studies within the region (Andrade-C et al., 2000; Andrade-C, 2010; Alcaldía Mayor de Bogotá, 2019; Durán Prieto & Montoya Quiroga, 2019), *G. drusilla*, *H. erato hydara* and *A. eurimedia* constitute new geographical distributional records for the city of Bogotá, D.C. These species are usually reported below 2000m elevation (DeVries, 1987; Le Crom et al., 2004; Andrade-C et al., 2007; Vargas-Zapatas et al., 2012). Insects in the tropical Andes may exhibit significant altitudinal migrations due to the effects of climate change (Colwell et al., 2008). For instance, species turnover and abundance of butterflies have been observed at elevations from 117 m to 3000 m in southeastern Mexico, with climate-induced changes recorded over a 23-year period (Colwell et al., 2008). Therefore, long-term monitoring of butterfly assemblages in the Bogotá region, considering the altitudinal gradients in high mountain ecosystems, is recommended to record potential impacts of climate change and land use conversion.

Comparing the number of species in the urban parks with the highest reported number of species in the peri-urban area of Bogotá (S=169, Henao-Bañol et al., 2018), our study reveals that urban parks within the city house only 7.10% of the species found in peripheral areas. If the three new records are included, the percentage rises to 8%. This suggests that most regional butterfly species have limited dispersal within the urban matrix. Although the study did not evaluate the effect of urbanization on butterfly diversity along a rural-peri-urban and urban gradient, it clearly demonstrates a reduction in butterfly richness within the urban area compared to rural and peri-urban areas, with most species being generalists. For sampling completeness, it is important to consider the timing of samplings, which can influence species presence based on environmental conditions (Jimenez-Valverde & Hortal, 2003). Therefore, sampling should be extended to other seasons. Furthermore, previous sampling efforts in Bogotá's urban parks have resulted in the collection of other species not included in this study, such as *Vanessa virginiensis* (Nymphalidae) and species from the Lycaenidae family (Andrade-C et al., 2000; Andrade-C, 2010).

Butterfly species in urban parks of Bogotá tended to be common and generalists displaying typical open area adapters. The species *L. aripa*, *L. eleone eleone*, *C. dimera*, *E. Salome Gaugamela*, *L. nemesis*, *T. xanthodice*, *D. glycera* are commonly associated with cow paddocks and pastures in high mountains ecosystems (DeVries, 1987; Le Crom et al., 2004; Andrade, 2010; Henao-Bañol et al., 2018). On the other hand, *V. carye*, *D. plexippus nigrippus*, *E. pallida*, *H. phyleus phyleus*, and *T. alector alector*, are considered rare and usually hard to sample in ecological studies due to their cryptic colourations, fast flight behaviour, and host plant specificity (Vélez & Salazar, 1991; Neild, 2008; González Montaña & Andrade-C, 2008; Henao-Bañol et al. 2018; Durán-Prieto & Molina-Fonseca, 2020). The fact that 10 out of the 15 species were rare or uncommon compared to more natural surroundings (Caicedo-Hernández et al., 2017; Henao-Bañol et al., 2018; Durán-Prieto & Molina-Fonseca, 2020), suggests that it might be possible to maintain and enrich urban areas at different scales to support the diversity assemblages of butterflies within the city.

**References**

Andrade-C, M. G., Amat-G, G., & Renjifo, J. (2000). Guía preliminar de insectos de Santafé de Bogotá y sus alrededores. Bogotá D.C. Departamento Técnico Administrativo del Medio Ambiente.

Andrade-C, M. G., Campos-Salazar, L. R., González-Montaña, L. A., Pulido-B, H. W. (2007). Santa María mariposas alas y color. Serie de Guías de Campo del Instituto de Ciencias Naturales No. 2. Instituto de Ciencias Naturales, Universidad Nacional de Colombia.

Andrade-C, M. G. (2010). Proyecto corredor borde norte de Bogotá fase 1, Capítulo 7: Mariposas. (Informe técnico). Bogotá D.C: Instituto de Estudios Urbanos, Universidad Nacional de Colombia, CAR, Academia Colombiana de Ciencias Exactas, Físicas y Naturales, UDCA.

Alcaldía Mayor de Bogotá. (2019). Vecinos inesperados: relatos de la fauna silvestre de Bogotá – Guía de campo. Secretaría de Cultura, Recreación y Deporte.

Caicedo-Hernández, J. C., Rodríguez-Villamil, D. R., Henao-Banol E. R., & Hernández-Schimdt, M. (2017). Mariposas y polillas diurnas de los parques urbanos el Virrey y Chicó y algunas de sus plantas hospederas. Field Guides, 1-8. <https://fieldguides.fieldmuseum.org/es/gu%C3%ADas/gu%C3%ADa/930>

Colwell, R. K., Brehm, G., Cardelús, C. L., Gilman, A. C., & Longinom, J. T. (2008). Global warming, elevational range shifts, and lowland biotic attrition in the wet tropics. Science, 322(5899), 258-261. <https://www.science.org/doi/abs/10.1126/science.1162547>

DeVries, P. J. (1987). The Butterflies of Costa Rica and their natural history: vol 1 (Papilionidae, Pieridae, Nymphalinae). Princeton University Press.

Durán-Prieto, J., & Molina-Fonseca, A. G. (2020). Urban colors: Butterflies (Lepidoptera: Papilionoidea) of Bogotá Region (Colombia). Biota colombiana, 21, 21-39. <https://doi.org/10.21068/c2020.v21n02a02>

González Montaña, L. A., Andrade-C, M. G. (2008). Diversidad y biogeografía preliminar de las mariposas saltarinas (Lepidoptera: Hesperiidae) de Colombia. Revista de la Academia Colombiana de ciencias exactas, físicas y naturales, 32, 421-424. <https://www.accefyn.com/revista/Vol_32/124/421-433.pdf>

Henao-Bañol, E. R., Páez, A., & Rodríguez-M, J. V. (2018). Inventario de mariposas diurnas (Lepidoptera: Hesperioidea-Papilionoidea) de la Reserva Forestal Productora Protectora de la cuenca alta del río Bogotá (RFPP-CARB). Boletín Científico Centro de Museos Museo de Historia Natural, 22(2), 144-171. <https://doi.org/10.17151/bccm.2018.22.2.11>

Jiménez-Valverde, A., & Hortal, J. (2003). Las curvas de acumulación de especies y la necesidad de evaluar la calidad de los inventarios biológicos. Revista Ibérica de Aracnología, 8, 151–161. <http://sea-entomologia.org/PDF/RIA_8/R08-024-151.pdf>

Le Crom, J. F., Constantino, L. M., & Salazar, J. A. (2004). Mariposas de Colombia. Pieridae. Carlec Ltda., Bogotá.

Neild, A. F. E. (2008). The Butterflies of Venezuela., Part 2: Nymphalidae (Acraeinae, Libytheinae, Nymphalinae, Ithomiinae, Morphinae). Meridian Publications.

Vargas-Zapatas, M. A., Prince-Chacón, S., Martínez-Hernández, N. J. (2012). Estructura poblacional de Heliconius erato hydara Hewitson, 1867 (Lepidoptera: Nymphalidae) en la reserva campesina la montaña (RCM), departamento del Atlántico, Colombia. Boletín de la Sociedad Entomológica Aragonesa (SEA), 51, 273-281. <http://sea-entomologia.org/PDF/Boletin51/273281BSEA51Heliconiusestructurapoblacional.pdf>

Vélez, J., & Salazar, J. (1991). Mariposas de Colombia. Villegas Editores.

**Appendix S6**

***Urban Butterfly Conservation Proposals***

Bogotá recognizes biodiversity conservation within the city in the year 2000 by the Land Use Plan (Plan de Ordenamiento Territorial - POT) in which the environmental determinants for the main ecological structure concept where incorporated and defined as part of the soil uses in the city (Andrade et al., 2013). The Main Ecological Structure is made up of key natural elements (e.g protected areas) to maintain the environmental services from which the urban and rural populations benefit (Plan de Ordenamiento Territorial, 2021). Throughout the “Policy for the Management of Biodiversity Conservation in the Capital City” (Política para la gestión de la conservación de la biodiversidad en el Distrito Capital) (Secretaría Distrital de Ambiente & Conservación Internacional, 2010), it was possible to identify the need to involve conservation management in urban settings at the Capital District. It’s been suggested that some green areas within the city might serve as structural and functional corridors for flora and fauna and connecting protected areas (which are part of the Main Ecological Structure) by implementing ecological restoration strategies and silvicultural enrichment in urban parks (Secretaría Distrital de Ambiente & Conservación Internacional, 2010). Consequently, the Easter Hills and the Bogotá River might be connected; but much more research is needed to identify the native species’ dispersion and how such corridors benefit biodiversity.

Organisms such as butterflies are used as an educational model within urban areas since it helps people relate with nature (Ramírez-Restrepo & MacGregor-Fors, 2017). Apparently, *D. glycera* is a species highly valued by the inhabitants of the city of Bogotá, where it is commonly known as the “espejito del curubo” (Alcaldía Mayor de Bogotá, 2019). It is worth mentioning that in schools such as the Gimnasio Moderno de Bogotá, a breeding program for this species has been implemented as a pedagogical strategy, whose purpose is to teach students about the life cycle of this insect and its importance in ecosystems (Alcaldía Mayor de Bogotá, 2019).

Taking into account the strategies and guidelines for biodiversity conservation in Bogotá’s urban areas mentioned, the results from this study highlight the importance of taking actions that contribute to butterfly diversity by: 1) planting and monitoring native floral species in spaces that occur in the road network (e.g. sidewalks and pedestrian paths), District parks (e.g. neighborhood and metropolitan parks), and in protected areas and ecological corridors (district ecological parks and ecological ring corridors) that comply with the regulations and guidelines given by “The Vegetable Cover Manual of Bogotá, D.C.” by the Jardín Botánico de Bogotá & Universidad de los Andes (2020); 2) promoting the use of manuals such as the books “Biodiverse Gardens for Bogotá” by Mahecha et al. (2020), and “Basic Steps to Stablish and Manage your Garden: A Practical Guide for Urban Farmers” by Herrera & Lara (2020), in this way, citizens may help to enrich the flora of gardens, roofs, green facades, and orchards to promote connectivity and increase quality of habitat for urban biodiversity; and 3) citizen science participation is needed to observe and monitor butterflies (van Tongeren et al., 2023), whether in parks or gardens, using guides such as “Guidelines for Standardized Global Butterfly Monitoring” by Van Swaay et al. (2015). For example, the Alexander von Humboldt Biological Resources Research Institute has compiled project experiences to exchange knowledge regarding actions that promote the conservation of urban biodiversity in Colombia (Díaz Arteaga et al., 2016). The proposal is aligned with the need to seek strategies in which people take ownership of the monitoring process of urban biodiversity and conservation of green areas to mitigate the impacts and transformation of the habitat of the species that generate a loss of biodiversity, ecosystem functions, and processes (e.g., Figueroa-Arango 2020).

**References**

Alcaldía Mayor de Bogotá. (2019). Vecinos inesperados: relatos de la fauna silvestre de Bogotá – Guía de campo. Secretaría de Cultura, Recreación y Deporte.

Andrade, G. I., Remolina, F., & Wiesner, D. (2013). Assembling the pieces: a framework for the integration of multi-functional ecological main structure in the emerging urban region of Bogotá, Colombia. Urban ecosystems, 16, 723-739. <https://doi.org/10.1007/s11252-013-0292-5>

Díaz Arteaga, A., Parra-Hinojosa, A., Zúñiga Bolíbar, A., Velásquez-Valencia, A., Cáceres-Franco, A. D. P., Saldaña Barahona, A. Y., ... & Mena García, Z. M. (2016). Naturaleza urbana. Plataforma de experiencias. In Mejía, M. A. (Ed.), Instituto de Investigación de Recursos Biológicos Alexander von Humboldt (pp. 5-7). Editorial Alexander von Humboldt.

Figueroa-Arango, C. (2020). Guía para la integración de las Soluciones Basadas en la Naturaleza en la planificación urbana: Primera aproximación para Colombia. Instituto de Investigación de Recursos Biológicos Alexander von Humboldt.

Herrera, G. E. G., & Lara, G. E. H. (2020). Pasos básicos para establecer y manejar tu huerta. Una guía práctica para agricultores urbanos. Jardín Botánico de Bogotá José Celestino Mutis. (1st ed). Ediciones Uniandes. <https://jbb.gov.co/documentos/cientifica/publicaciones/Pasos_basicos_para_establecer_y_manejar_tu_huerta.pdf>

Jardín Botánico de Bogotá & Universidad de los Andes. (2020). El Manual de Coberturas Vegetales de Bogotá, D.C. (1st ed.). Ediciones Uniandes. <https://jbb.gov.co/documentos/cientifica/publicaciones/MANUAL_COBERTURAS_VEGETALES.pdf>

Mahecha, G.E., Duque-Osorio, R., Morales-Liscano, G., Serrano, C. M., & Torres, M. E. (2020). Jardines Biodiversos para Bogotá. Jardín Botánico de Bogotá.

Plan de Ordenamiento Territorial. (2021). Proyecto de acuerdo Plan de Ordenamiento Territorial: Bogotá Reverdece 2022-2035. <https://bogotacomovamos.org/proyecto-de-acuerdo-pot-bogota-2022-2035/>

Ramírez-Restrepo, L., & MacGregor-Fors, I. (2017). Butterflies in the city: a review of urban diurnal Lepidoptera. Urban Ecosystems, 20, 171-182. <https://doi.org/10.1007/s11252-016-0579-4>

Secretaría Distrital de Ambiente & Conservación Internacional. (2010). Política para la Gestión de la Conservación de la Biodiversidad en el Distrito Capital. Editorial Panamericana.

van Tongeren, E., Sistri, G., Bonifacino, M., Menchetti, M., Pasquali, L., Salvati, V., Balleto, E., Bonelli, S., Cini, A., Portera, M., & Dapporto, L. (2023). Unstructured citizen science reduces the perception of butterfly local extinctions: the interplay between species traits and user effort. Biodiversity and Conservation, 32, 4701-4718. <https://doi.org/10.1007/s10531-023-02721-9>

Van Swaay, C., Regan, E., Ling, M., Bozhinovska, E., Fernandez, M., Marini-Filho, O. J., Huertas B., Phon, C. K., K”orösi, A., Meerman, J., Pe’er, G., Uehara-Prado, M., Sáfián, S., Sam, L., Shuey, J., Taron, D., Terblanche, R., & Underhill, L. (2015). Guidelines for Standardized Global Butterfly Monitoring. Group on Earth Observations Biodiversity Observation Network. GEO BON Technical Series 1. <https://www.geobon.org/downloads/biodiversity-monitoring/technical-reports/GEOBON/2015/Global-Butterfly-Monitoring-Print.pdf>

**Figures Supplementary Information**


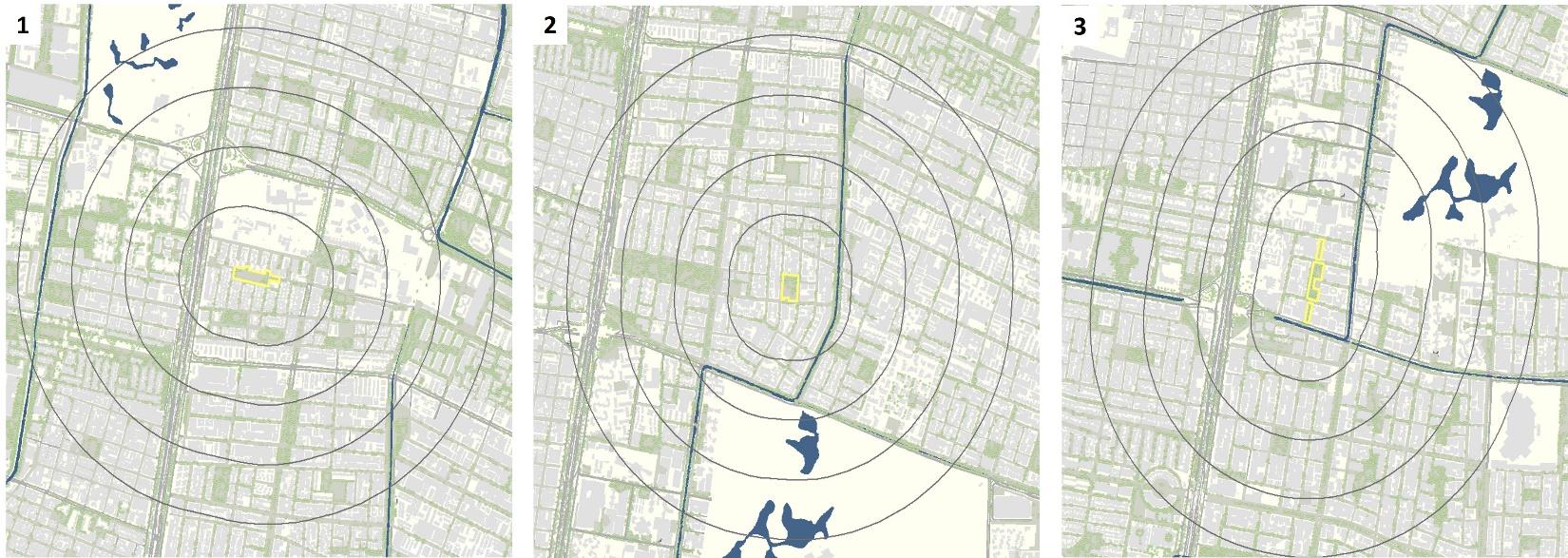


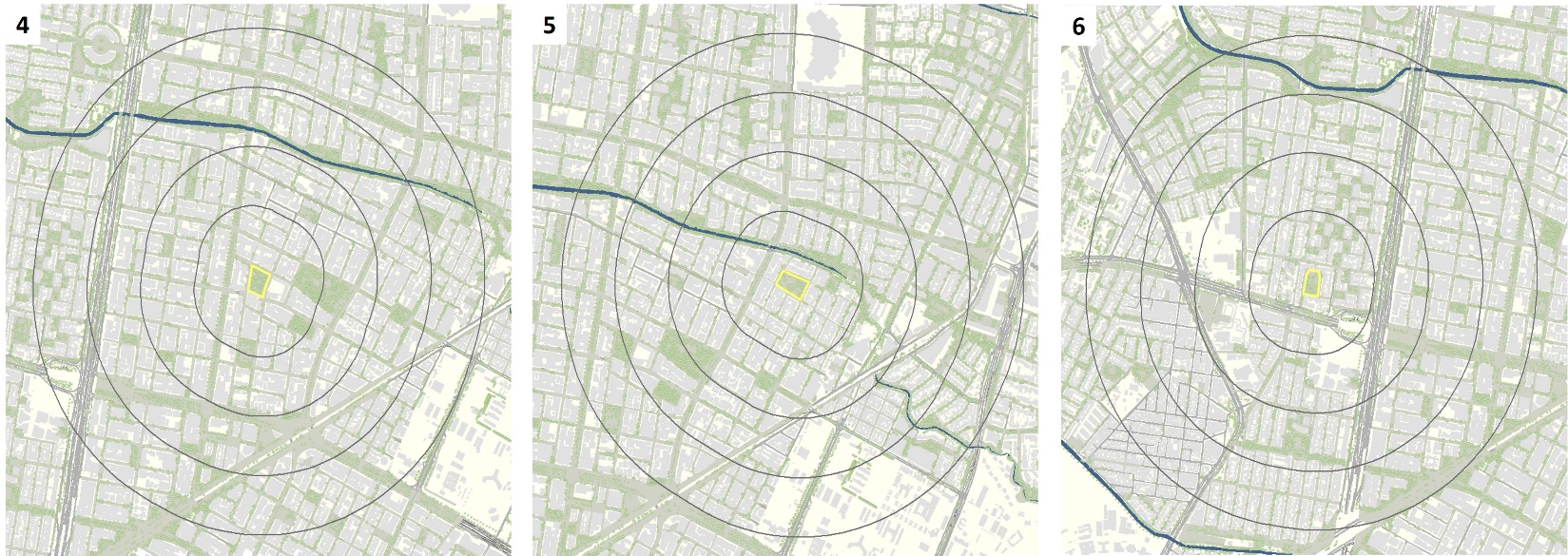


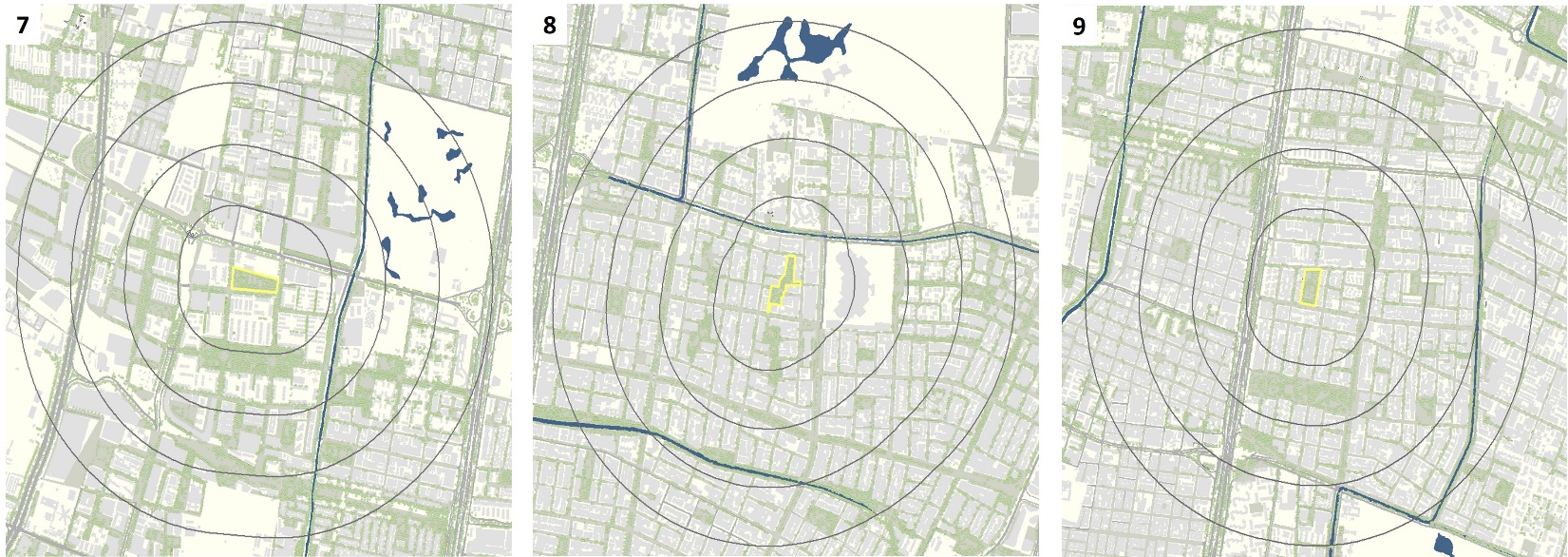


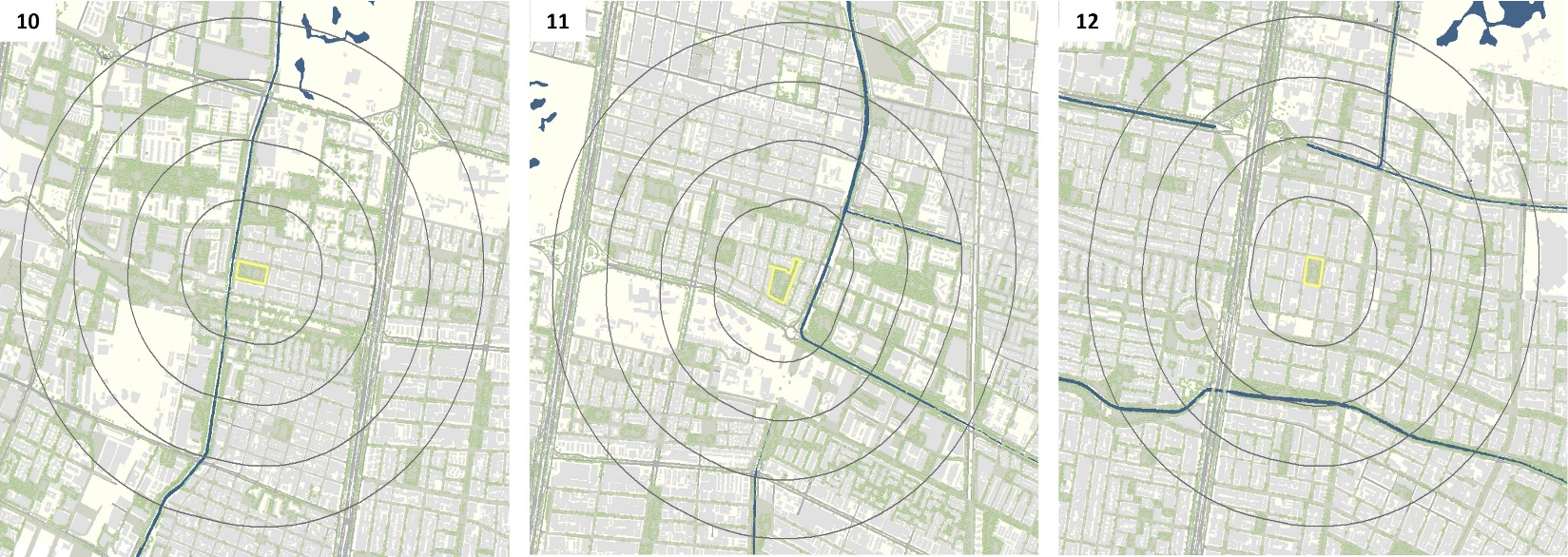


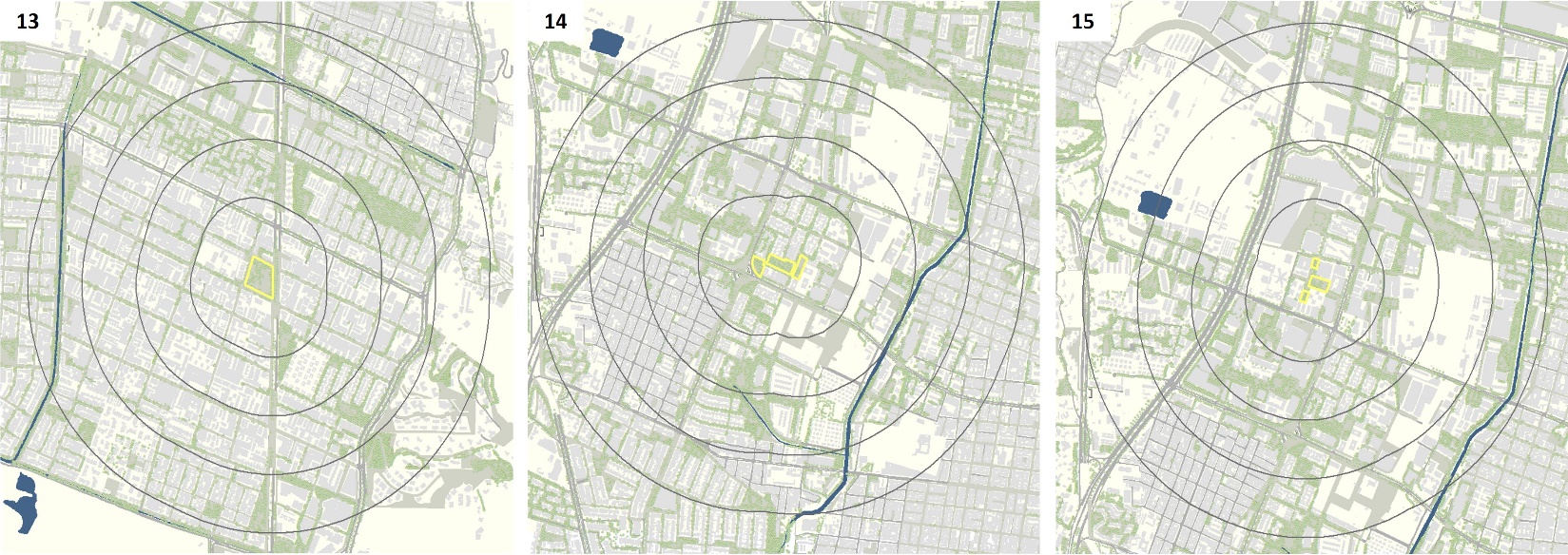


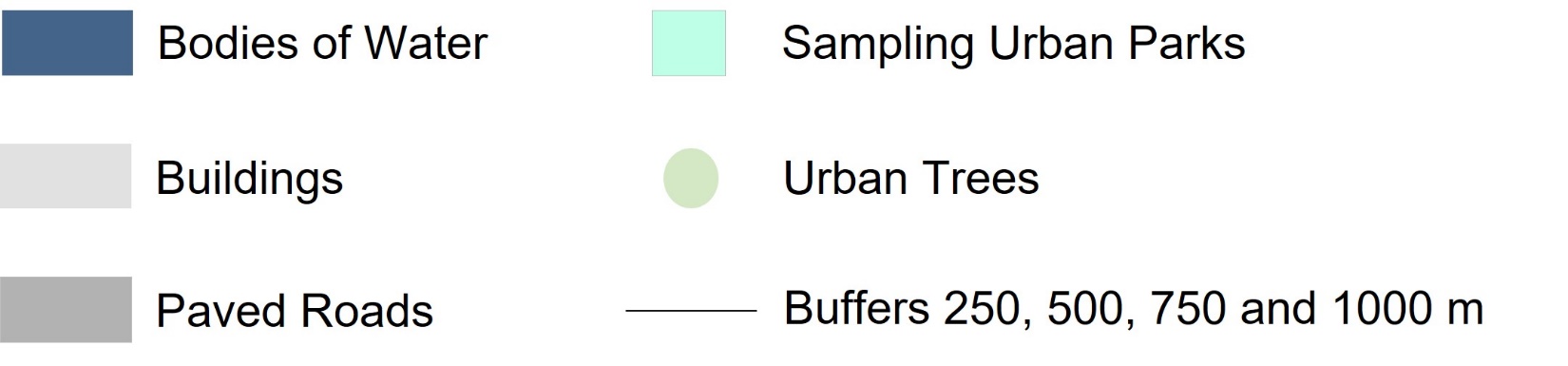


**Figure S1.** Neighborhood parks in the localities of Suba and Usaquén, Bogotá, DC (Colombia) with their buffer areas: local area (sampled parks, highlighted in yellow), and buffer zones at 250 m, 500 m, 750 m, and 1000 m (gray). 1= Cedro Bolívar (Usaquén), 2= El Contador (Usaquén), 3= La Calleja (Usaquén), 4= Navarra (Usaquén), 5= Santa Paula (Usaquén), 6= Urbanización Calle 100 (Suba), 7= Urbanización Versalles (Suba), 8= Santa Bárbara-1 (Usaquén), 9= Autopista Norte II Sector (Usaquén), 10= Victoria Norte (Suba), 11= Villa Magdala (Usaquén), 12= Santa Bárbara-2 (Usaquén), 13= El Cedrito (Usaquén), 14= Colina Norte II Etapa (Suba), and 15= Ciudad Privada Atenas (Suba). Layers obtained from Open Data of Bogotá D.C. (2022). SCR: CTM12, Projection: Transverse Mercator, 1:4200.


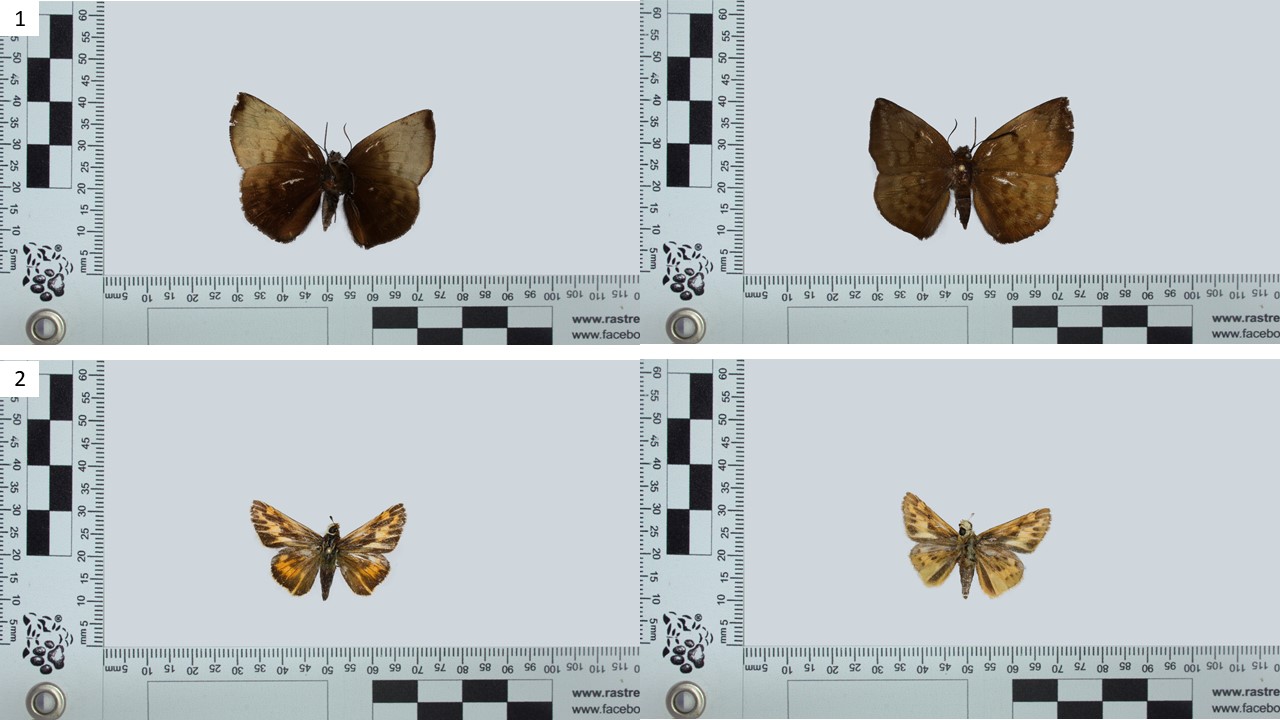


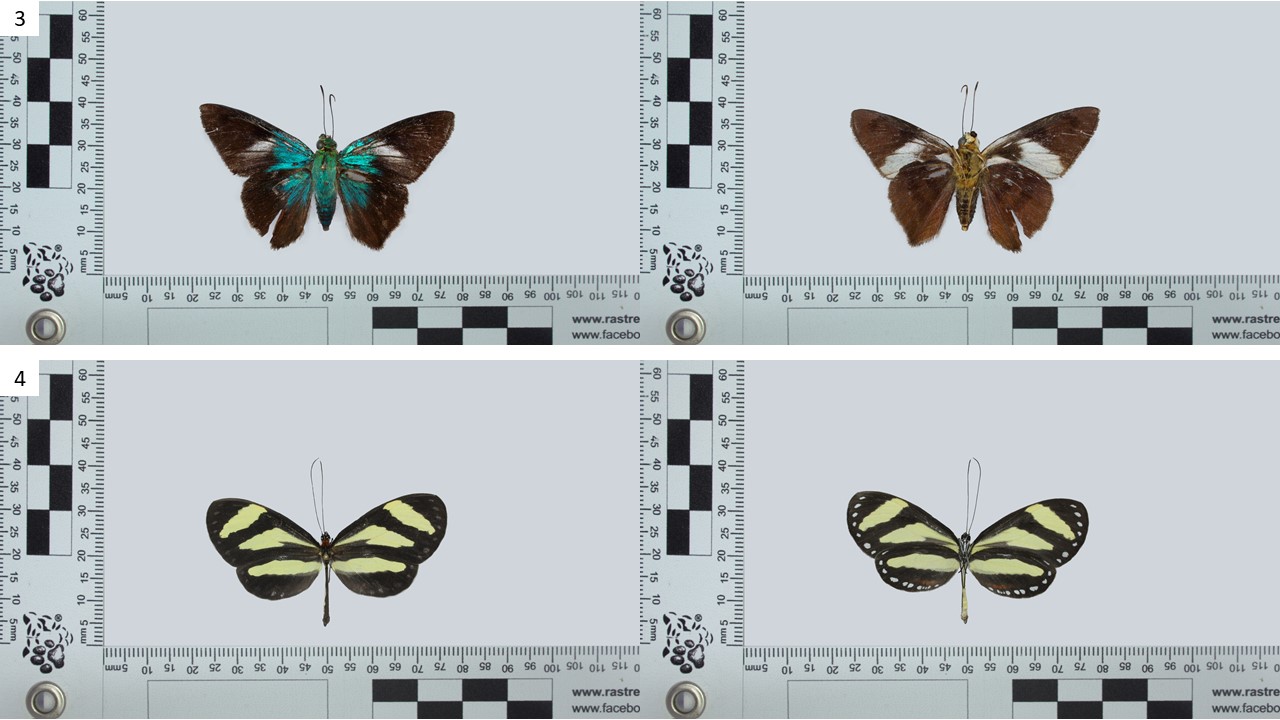


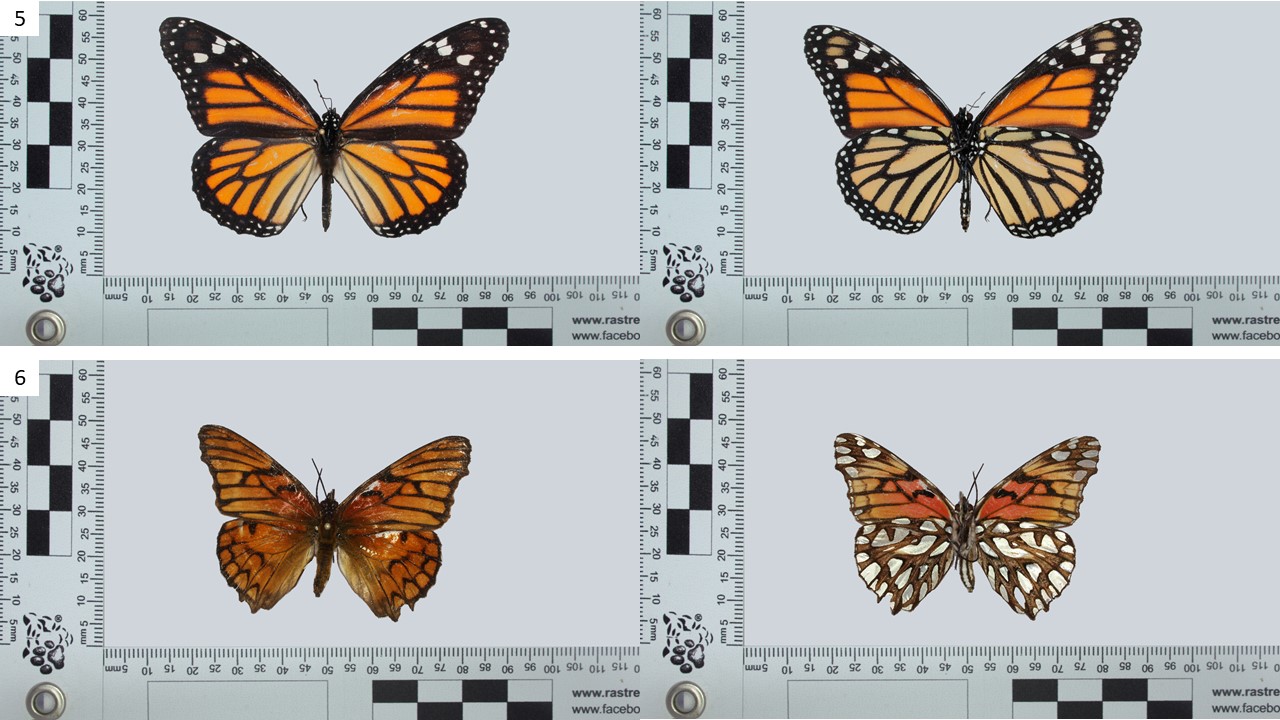


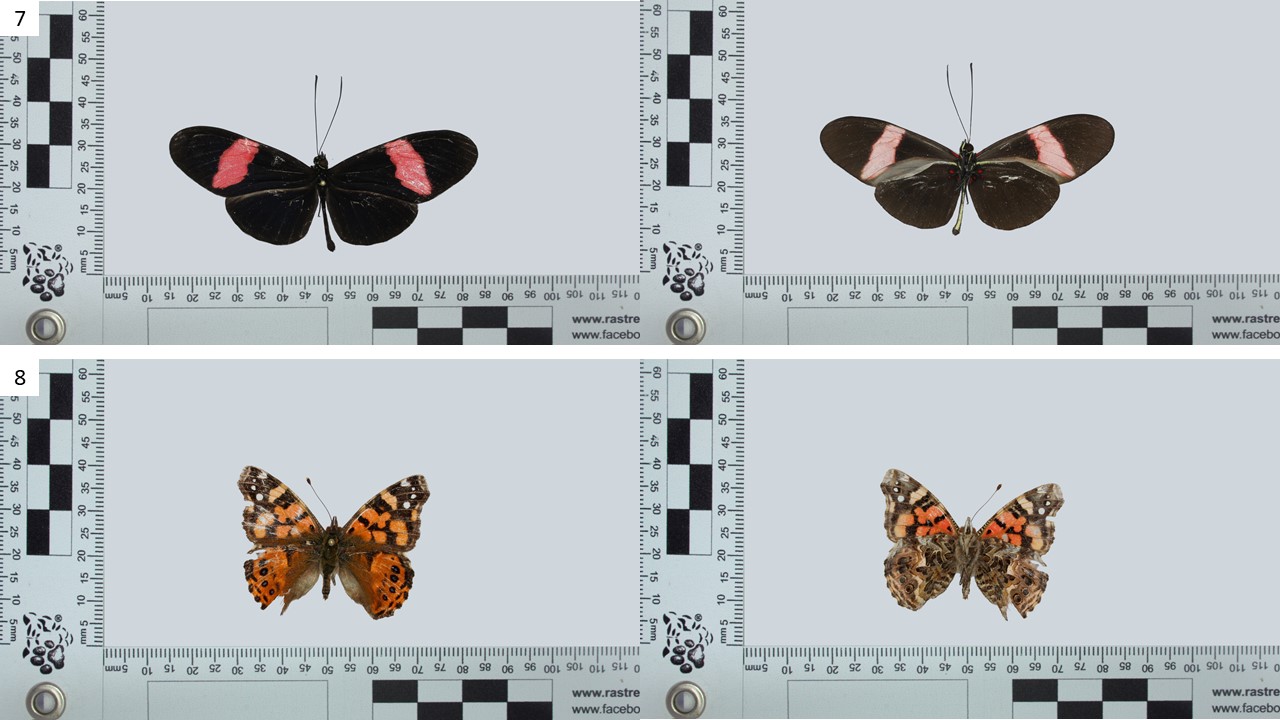


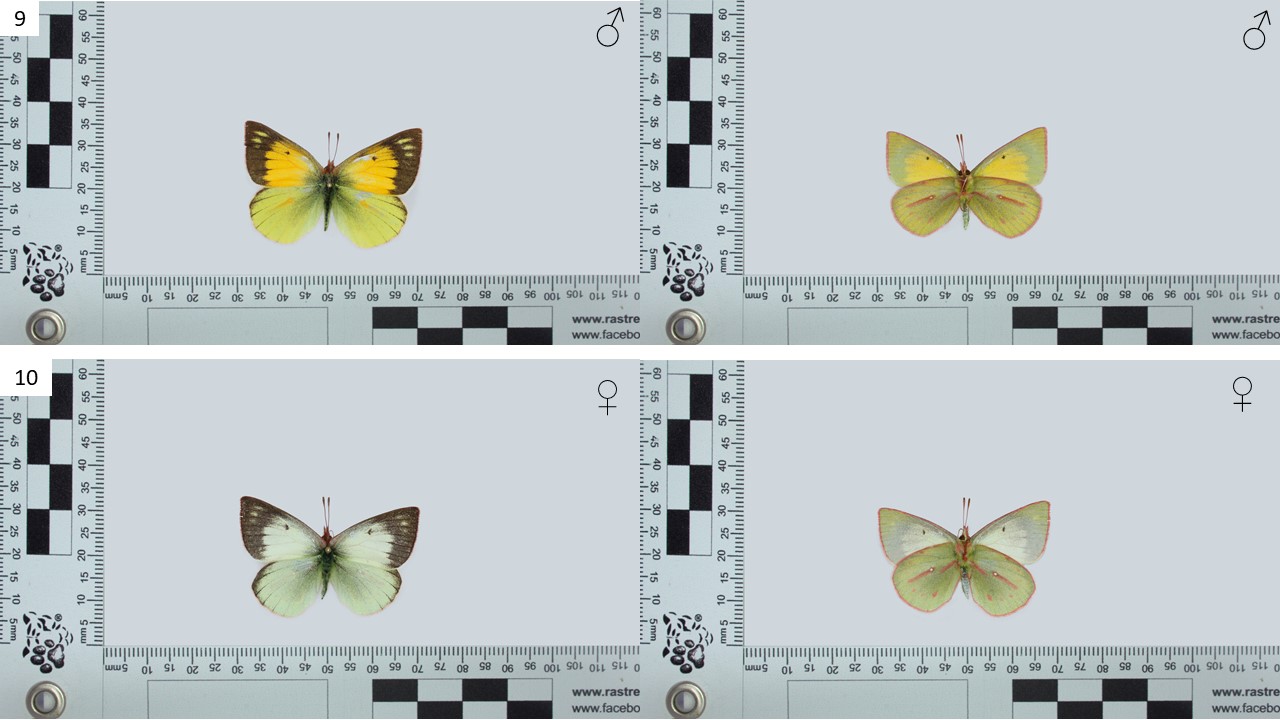


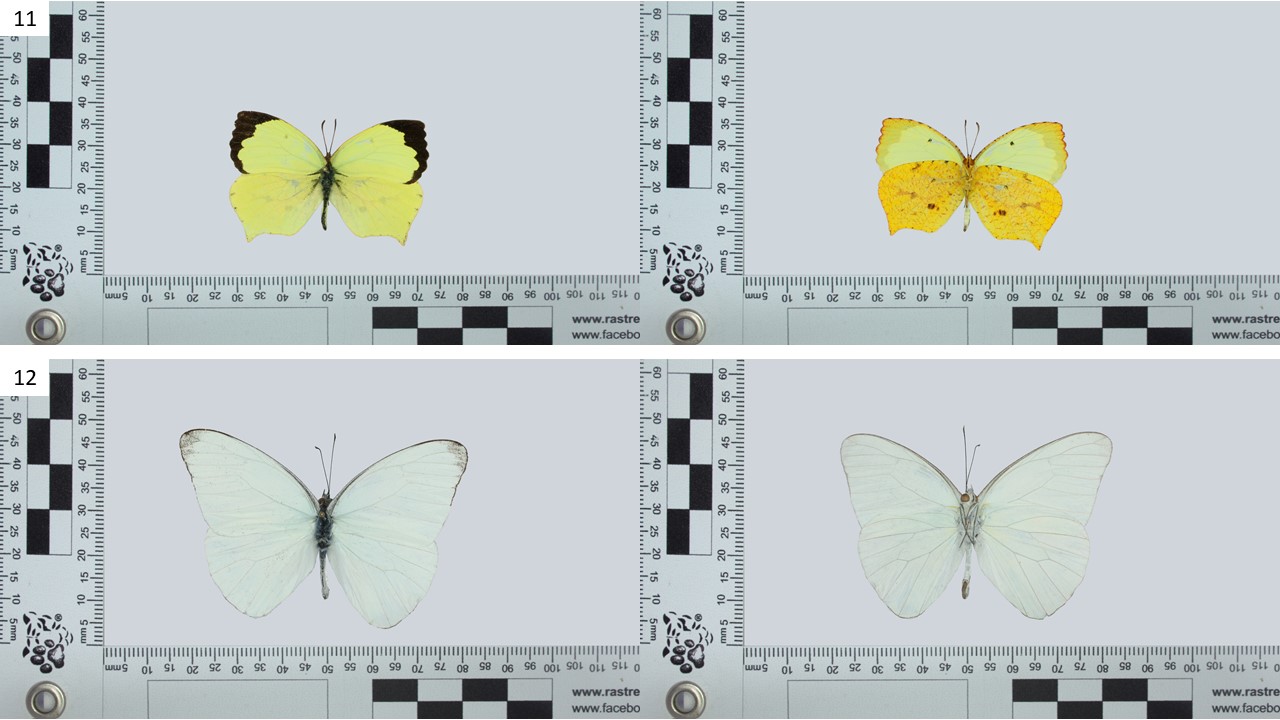


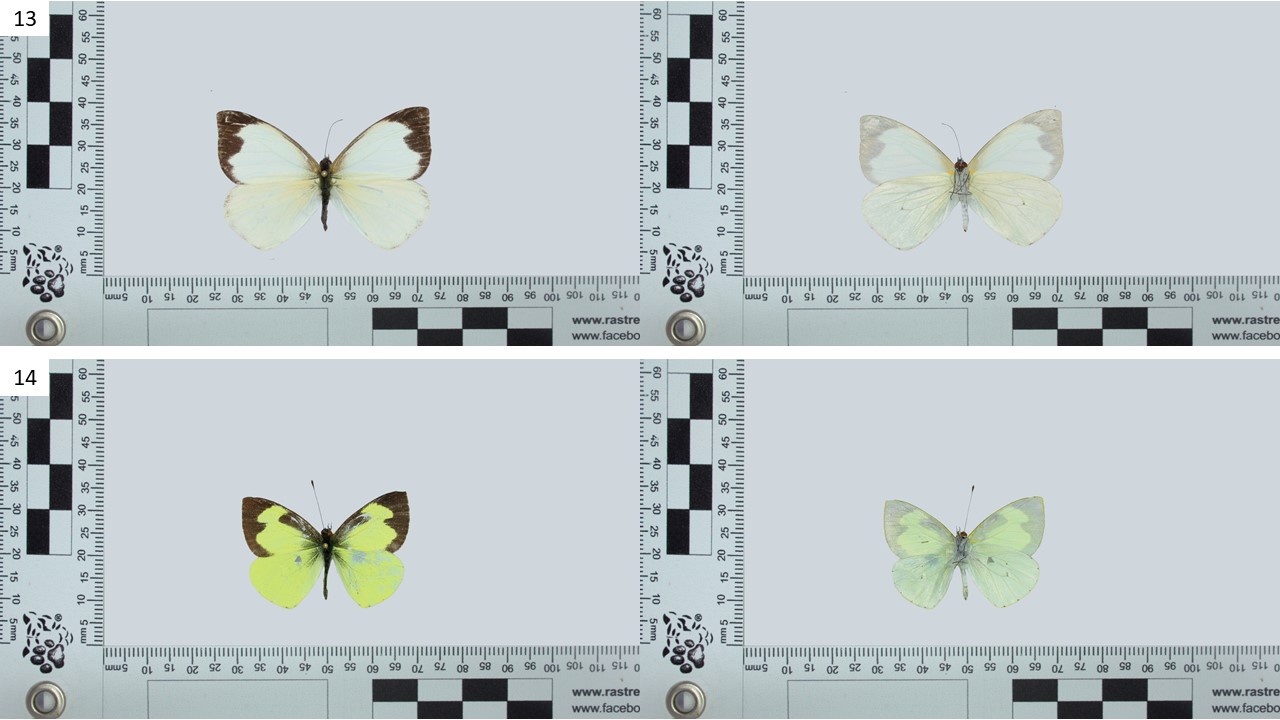


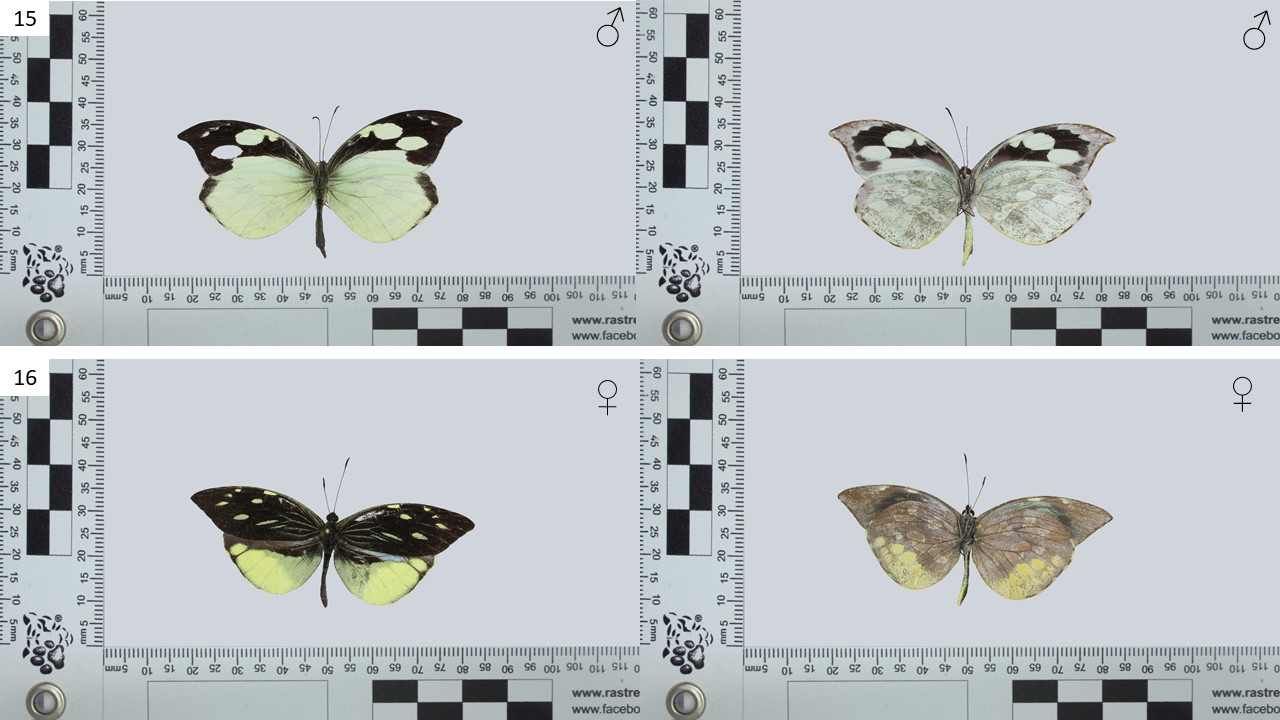


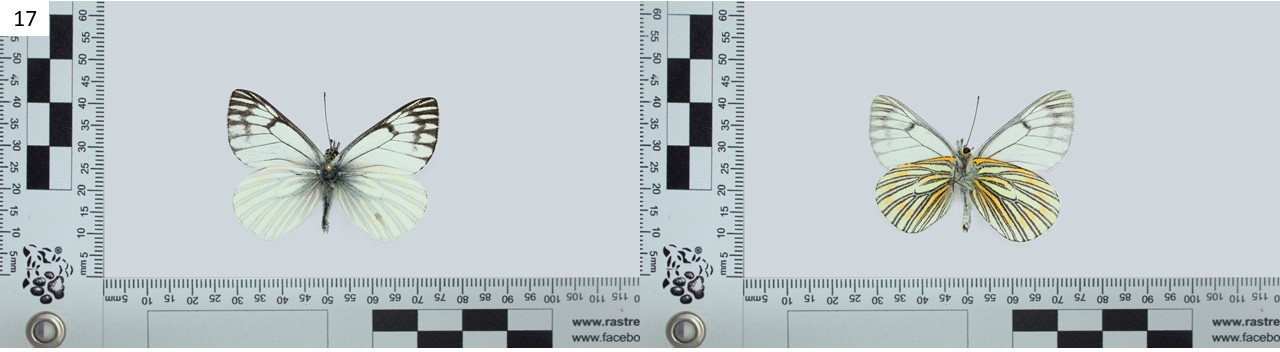


**Figure S2.** Photos of the 15 species of butterflies. Family Hesperiidae: 1=*Eantis pallida*, 2=*Hylephila phyleus phyleus* and 3=*Telegonus alector alector*. Family Nymphalidae: 4= *Aeria eurymedia*, 5= *Danaus plexippus nigrippus*, 6= *Dione glycera*, 7= *Heliconius herato hydara* and 8= *Vanessa carye*. Family Pieridae: 9= *Colias dimera* (male), 10= *Colias dimera* (female), 11= *Eurema salome gaugamela*, 12=*Glutophrissa drusilla drusilla*, 13= *Leptophobia aripa aripa*, 14= *Leptophobia eleone eleone*, 15= *Lieinix nemesis* (male), 16= *Lieinix nemesis* (female) and 17= *Tatochila xanthodice*.


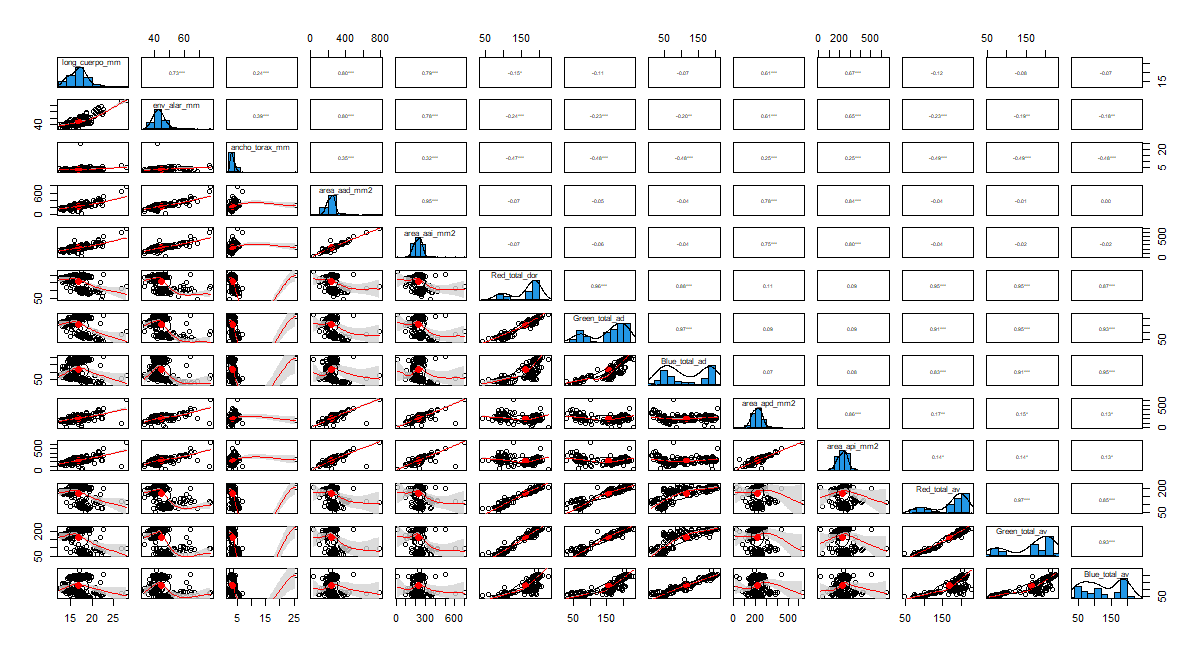

**Figure S3.** Spearman Correlation Analysis of 13 Functional Traits in 268 Butterflies: Selection Criteria Excluding Variables Correlating with More Than 5 Traits Above 80%, Resulting in 5 Traits. Thorax Width Showed No Correlation, and Only Wing Length and Span Had Correlations Below 80%, with Wing Span Providing Better Ecological Interpretations.
